# Metabolome combined with transcriptome profiling reveals the dynamic changes in flavonoids in red and green leaves of *Populus* × *euramericana* ‘Zhonghuahongye’

**DOI:** 10.3389/fpls.2023.1274700

**Published:** 2023-12-21

**Authors:** Yun Yang, Mengjiao Chen, Wan Zhang, Haiyang Zhu, Hui Li, Xinjiang Niu, Zongshun Zhou, Xiaoya Hou, Jingle Zhu

**Affiliations:** ^1^ Research Institute of Non-Timber Forestry, Chinese Academy of Forestry, Zhengzhou, Henan, China; ^2^ Key Laboratory of Non-timber Forest Germplasm Enhancement and Utilization of National Forestry and Grassland Administration, Zhengzhou, Henan, China; ^3^ Research Institute of Tropical Forestry, Chinese Academy of Forestry, Guangzhou, Guangdong, China; ^4^ College of Forestry, Henan Agricultural University, Zhengzhou, Henan, China; ^5^ Institute of Gene Science and Industrialization for Bamboo and Rattan Resources, International Center for Bamboo and Rattan, Beijing, China; ^6^ China Experimental Centre of Subtropical Forestry, Chinese Academy of Forestry, Xinyu, Jiangxi, China

**Keywords:** *Populus × euramericana* ‘Zhonghuahongye’, flavonoids, anthocyanin, metabolome, RNA-seq

## Abstract

Flavonoids are secondary metabolites that have economic value and are essential for health. Poplar is a model perennial woody tree that is often used to study the regulatory mechanisms of flavonoid synthesis. We used a poplar bud mutant, the red leaf poplar variety 2025 (*Populus × euramericana* ‘Zhonghuahongye’), and green leaves as study materials and selected three stages of leaf color changes for evaluation. Phenotypic and biochemical analyses showed that the total flavonoid, polyphenol, and anthocyanin contents of red leaves were higher than those of green leaves in the first stage, and the young and tender leaves of the red leaf variety had higher antioxidant activity. The analyses of widely targeted metabolites identified a total of 273 flavonoid metabolites (114 flavones, 41 flavonols, 34 flavonoids, 25 flavanones, 21 anthocyanins, 18 polyphenols, 15 isoflavones, and 5 proanthocyanidins). The greatest difference among the metabolites was found in the first stage. Most flavonoids accumulated in red leaves, and eight anthocyanin compounds contributed to red leaf coloration. A comprehensive metabolomic analysis based on RNA-seq showed that most genes in the flavonoid and anthocyanin biosynthetic pathways were differentially expressed in the two types of leaves. The flavonoid synthesis genes *CHS* (chalcone synthase gene), *FLS* (flavonol synthase gene), *ANS* (anthocyanidin synthase gene), and proanthocyanidin synthesis gene *LAR* (leucoanthocyanidin reductase gene) might play key roles in the differences in flavonoid metabolism. A correlation analysis of core metabolites and genes revealed several candidate regulators of flavonoid and anthocyanin biosynthesis, including five MYB (MYB domain), three bHLH (basic helix-loop-helix), and HY5 (elongated hypocotyl 5) transcription factors. This study provides a reference for the identification and utilization of flavonoid bioactive components in red-leaf poplar and improves the understanding of the differences in metabolism and gene expression between red and green leaves at different developmental stages.

## 1 Introduction

Poplars (*Populus*, *Salicaceae*) have strong regeneration ability and wide-ranging economic and ecological benefits (Koes et al., 2005; Felix et al., 2008). There are 35 tree species of the genus *Populus*, including *P. alba*, *P. × canescens*, *P. nigra*, *P. tremula*, *P. tremuloides*, and *P. deltoides*. Poplar is a rich source of phytocompounds, including proteins, vitamins, mineral elements, phenolic compounds, and terpenoids, flavonoids (Devappa et al., 2015). Research on poplars has mainly focused on the cultivation of new varieties and the development of planting technologies (Biselli et al., 2022), while research on the utilization of poplars as phytochemical resources is limited. The potential applications of poplar extract might provide new opportunities in the pharmaceutical industry (Guleria et al., 2022). In addition, with the development of feed processing technology, research on poplar leaves as feed additives has increased (McAvoy et al., 2020). As early as 1980, Canadian scientists assumed the cultivation of a ruminant-type animal that feeds on poplar leaves. Recent studies have shown that the use of poplar leaves in appropriate amounts as feed additives can reduce methane emissions and improve the antioxidant activity of blood in buffalo (*Bubalus bubalis*) calves (Dey et al., 2021; Kumar et al., 2022).

Chemical extraction and isolation of metabolites from poplars are performed mainly by chromatographic separation and structural elucidation techniques, including high-performance liquid chromatography (HPLC), nuclear magnetic resonance (NMR), and liquid chromatography tandem mass spectrometry (LC−MS) (Guleria et al., 2022). Over 155 specific metabolites, including 27 phenolic acids and their derivatives, 32 phenolic glycosides, 53 flavonoids, and 44 terpenoids, have been characterized from poplar plants (Kis et al., 2020). Among all bioactive ingredients, flavonoids have antioxidant, anti-inflammatory, antibiotic, and anticarcinogenic potential (Panche et al., 2016). Flavones, flavonols, flavanones, flavanonols, isoflavones, catechins, anthocyanins, and proanthocyanidins are subgroups of flavonoids (Panche et al., 2016). The anthocyanins and flavonoids separated from *Delonix regia* flowers inhibited the activity of pathogenic bacteria *in vitro* (Ebada et al., 2023). *Morus alba* L. leaf extracts were studied for clinical treatment of diabetes and obesity because they are rich in isoquercitrin, kaempferol, quercetin, rutin, chlorogenic acid, and gallic acid (Zhang et al., 2022). Bud extracts of *P. nigra*, with the bioactive ingredients caffeic and p-coumaric acids, have high antioxidant potential (Dudonné et al., 2011). Therefore, it is important to accurately identify the flavonoid metabolites in poplars to effectively use these resources.

Plant flavonoid metabolites and their distribution are important subjects and hot topics for research (Shen et al., 2022b). Although flavonoid biosynthesis pathways are dependent on plant species and environmental conditions, the development of plant or microbial cell engineering and genetic engineering provides the possibility of industrial biosynthesis of specific flavonoids (Shen et al., 2022a). Flavonoid regulation has the same upstream chemical process as proanthocyanidin and anthocyanin biosynthesis (Falcone Ferreyra et al., 2012; Liu et al., 2021). MYB, basic helix-loop-helix (bHLH), and WD40 transcription factors (TFs) play an important role in regulating flavonoid synthesis (LaFountain and Yuan, 2021). TFs can promote or suppress flavonoid synthesis by recognizing and binding specific sequences of structural gene promoters (An et al., 2012; Zhu et al., 2015). There are many TFs in poplar that might be related to flavonoids, and these await further exploration.

*Populus* × *euramericana* ‘Zhonghuahongye’ is a bud mutant that was selected from *Populus nigra* L2025 (green leaves) (Chen et al., 2022a). This variety is widely planted commercially for landscaping. The color of *Populus* × *euramericana* ‘Zhonghuahongye’ leaves varies from bright red to reddish brown with age. Flavonoid and anthocyanin metabolites are differentially expressed as the color changes (Chen et al., 2023). Another study showed that in *Populus deltoides* varieties with red leaves, the contents of quercetin, rhamnetin, isorhamnetin, and kaempferol were higher than those in the green leaves of *Populus* sp. Linn. ‘2025’ (Tian et al., 2021). Red poplar varieties have higher feeding value than common poplar varieties because of the flavonoid content (Ayers et al., 1996). There is currently no comprehensive and detailed description of whether the contents of these flavonoids vary among different growth stages of red poplar. What are the differences in flavonoids between red leaves and green leaves of *Populus nigra* over time? Which metabolic pathways and putative genes contribute to these differences? In this study, *Populus* × *euramericana* ‘Zhonghuahongye’ leaves (red) and green leaves from three developmental stages were taken as the research material. Anthocyanins, flavanones, flavones, flavonoids, flavonols, isoflavones, polyphenols, and proanthocyanidins were identified. A comprehensive analysis was performed, and dynamic flavonoid accumulation in red leaves was observed. Structural gene expression and TF regulation provide the foundation for studying flavonoid biosynthesis. Our results provide insights into flavonoid metabolism in *Populus nigra* at different developmental stages and will be beneficial for the utilization of its leaf resources.

## 2 Materials and methods

### 2.1 Plant materials and sampling

**Figure 1:**
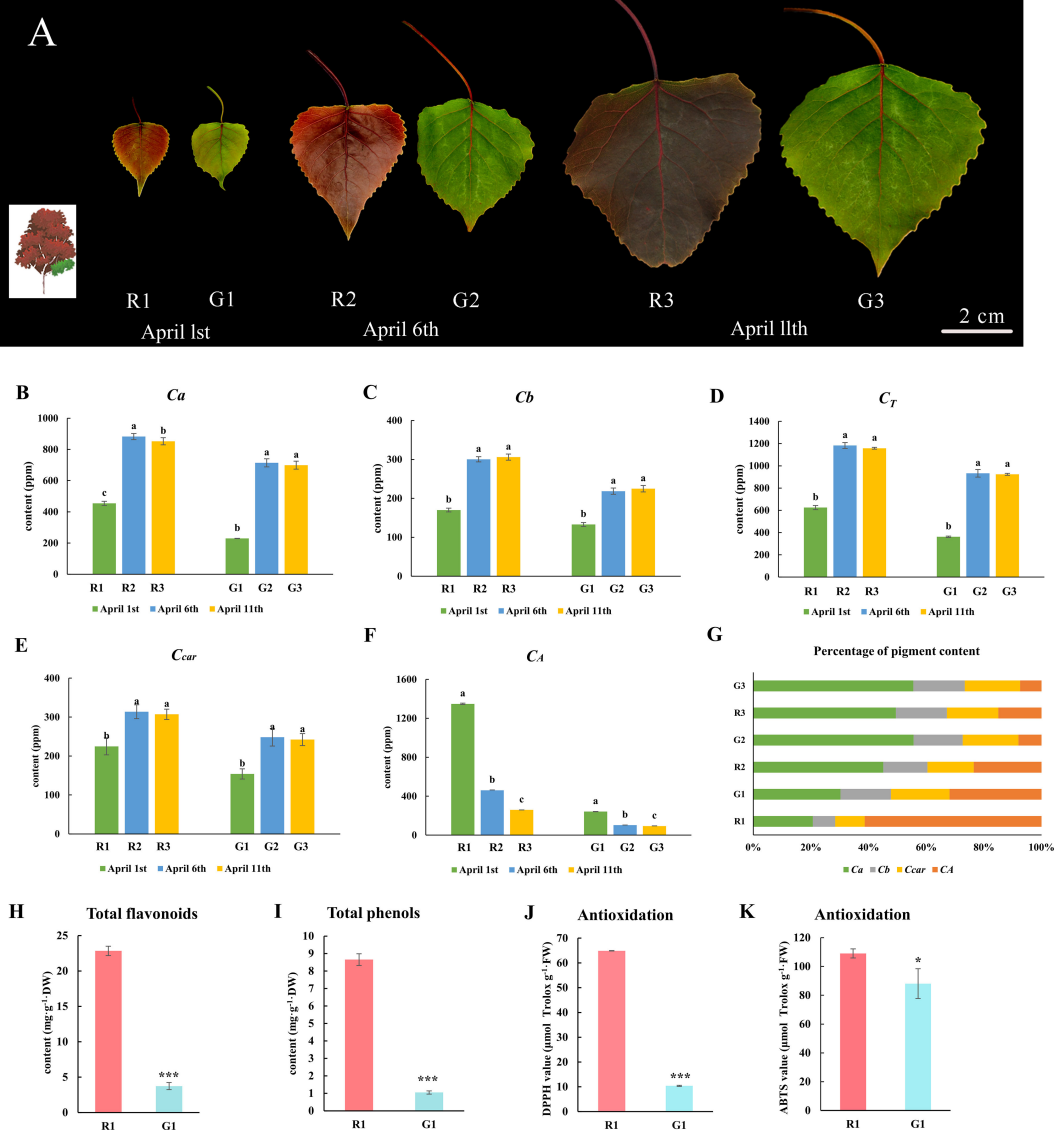
Morphology, pigment content, total flavonoid content and polyphenol content, and antioxidant ability of green and red poplar leaves. **(A)** Morphological observations of red and green leaves on 1 April 2019, 6 April 2019, and 11 April 2019; **(B)** chlorophyll a content; **(C)** chlorophyll b content; **(D)** total chlorophyll content; **(E)** carotenoid content; **(F)** anthocyanin content; **(G)** percentage of pigment content in each sample. These data were analyzed using Duncan’s multiple range test (*p-*value ≤ 0.05) and are expressed as the means ± standard deviations (SDs) of triplicates. **(H)** Total flavonoid content; **(I)** total polyphenol content; **(J)** DPPH values; **(K)** ABTS values. These data of R1 and G1 leaves were analyzed by the T-test method. *** indicates a significant *p* value ≤ 0.001, and * indicates a *p* value ≤ 0.05. The small letters represent significant differences at three different developmental stages.

The *Populus* × *euramericana* ‘Zhonghuahongye’ variety is a bud mutant of the Poplar L2025 clone. The *Populus* × *euramericana* ‘Zhonghuahongye’ variety is a highly valuable ornamental tree that exhibits red leaves throughout the growth cycle. From spring to early summer, all the plant leaves, especially newly sprouted leaves, are a beautiful rose-red color. In the middle stage of growth, the leaves are bright purplish-red, and the mature leaves turn red−green. The studied plants were kept at the state-owned Mengzhou exploratory site (north latitude 32°06’; east longitude 118°06’). However, several branches with green leaves were found on the red-leaved trees. Thus, green leaves and red leaves of 3-year-old trees were collected for the experiment. Red leaves were collected at three stages: R1 (1 April 2019), R2 (6 April 2019), and R3 (11 April 2019). Green leaves were collected on the same dates and referred to as the G1, G2, and G3 samples (**Figure 1A**). Three trees were selected as biological replicates. For each sample, leaves with similar positions and colors on the branches were selected. The sampling procedures are shown in **Figure S1**.

### 2.2 Leaf color measurement and determination of physiological indexes

Leaf color was measured according to the International Commission on Illumination (CII) color standard. L*, a*, and b* values were determined using a CR2500 chromatic aberration meter (Minolta, Japan). Ten points were randomly selected on each blade, chromatic aberration timing was used to avoid the leaf veins, and the average value for each blade was used for analysis (not transparent when measuring). The measured a*, b*, and L* values could be used to calculate chromaticity (C*) and light color values (Tian et al., 2021; Yang et al., 2021). The photosynthetic pigment contents of the R1, R2, R3, G1, G2, and G3 samples were determined. The chlorophyll a (C_a_), chlorophyll b (C_b_), total chlorophyll (C_T_), carotenoid (C_car_), and anthocyanin (C_A_) contents were determined by ultraviolet spectrophotometry (JINGHUA Instruments 752), and 3 biological replicates were analyzed per sample. The experiment was performed according to a published paper (Chen et al., 2022a). The data were analyzed by SPSS 17.0 software.

The leaves were dried to a constant weight. Then, the samples were precisely weighed to 0.5000 g, extracted with 25 mL of boiling water for 5 min, heated in a 25°C water bath for 30 min, and centrifuged at 10,000 r · min ^-1^ for 10 min, after which the precipitate was discarded. The supernatant was used for further analysis. The total flavonoid content was determined by the AlCl_3_ - (HAc - NaAc) chromogenic method (Han et al., 2014). Rutin was used as the standard, and the absorbance was measured at 400 nm. The total phenol content was determined spectrophotometrically by the Folin-Ciocalteu method (Han et al., 2015). Gallic acid was used as the standard, and the absorbance was measured at 765 nm. In addition, the extraction method used for fresh samples was the same as that used for dry samples. The DPPH assay and ABTS^+^ assay for studying free radical scavenging activity were performed using fresh samples. DPPH solution exhibits maximum absorption at 517 nm. The procedure was as follows: 1 mL of DPPH solution (0.05 g · mL^-1^) was prepared, 500 μL of sample solution was added, and the mixture was reacted at room temperature for 30 min before measurement. ABTS^+^ solution exhibits maximum absorption at 734 nm. In the assay, 300 μL of sample solution and 1.2 mL of ABTS^+^ application solution was mixed evenly. The mixture was allowed to stand in the dark for 6 min and then measured. Trolox was used as a standard solution to quantify the antioxidant capacity.

### 2.3 Metabolite data analysis

Sample pretreatment and metabolite detection and quantification were accomplished at Wuhan MetWare Biotechnology Co., Ltd. (www.metware.cn) according to a standard procedure. Leaf metabolites were extracted from 100 mg powdered samples in 70% aqueous methanol (1.0 mL) for 24 h at 4°C. Following centrifugation at 10,000 × g for 10 min, the extracts were absorbed (CNWBOND Carbon-GCB SPE Cartridge, 250 mg, 3 mL; ANPEL, Shanghai, China) and filtered (SCAA-104, 0.22 μm pore size; ANPEL, Shanghai, China) before LC-MS analysis.

The sample extracts were analyzed using an LC-ESI-MS/MS system (HPLC, Shim-pack UFLC SHIMADZU CBM30A system, MS; Applied Biosystems 4500 Q TRAP). The analytical conditions were described in **Table S1**. The effluent was alternatively directed to an ESI-triple quadrupole-linear ion trap (Q TRAP)-MS. LIT and triple quadrupole (QQQ) scans were acquired on a triple quadrupole-linear ion trap mass spectrometer (Q TRAP), an API 4500 Q TRAP LC/MS/MS System, equipped with an ESI Turbo Ion-Spray interface, operating in positive ion mode and controlled by Analyst 1.6.3 software (AB Sciex). The ESI-Q TRAP-MS/MS parameter is displayed in **Table S2**. A specific set of multiple reaction monitoring (MRM) transitions was monitored for each period according to the metabolites eluted within this period.

Analyst 1.6.3 software was used to process the mass spectral data. Based on the self-built database MWDB and the metabolite information public database, qualitative analysis of the primary and secondary mass spectral data was performed. The mass spectral peaks detected in different samples for each metabolite were corrected to ensure qualitative and quantitative accuracy (Fraga et al., 2010). Quality control (QC) of mixed samples was performed after every 10 samples prior to data analysis to monitor the reproducibility of the analytical process. QC samples were prepared using mixed sample extracts.

Orthogonal partial least squares discriminant analysis (OPLS-DA) and principal component analysis (PCA) were conducted to analyze and verify the differences and reliability of metabolites in the samples. The metabolite content data were normalized by the range method, and the accumulation mode of metabolites among different samples was analyzed by heatmap cluster analysis (hierarchical cluster analysis, HCA) through R software (www.r-project.org/). PLS-DA analysis was applied to calculate the corresponding variable importance in projection (VIP) value. Metabolites with VIP≥ 1 and fold change ≥2 or fold change ≤ 0.5 were considered differentially expressed metabolites (DEMs). Then, the differentially abundant metabolites were mapped to the Kyoto Encyclopedia of Genes and Genomes (KEGG) database for significant enrichment analysis, and major enriched pathways were identified.

### 2.4 Transcriptomics

Eighteen sample libraries representing 2 leaf colors and 3 developmental stages were constructed for RNA-seq. The procedures for high-throughput sequencing were reported in previous studies (Chen et al., 2022a). An Illumina HiSeq instrument was used for sequencing. The *Populus trichocarpa* genome was selected as the reference (Tuskan et al., 2006). To quantify transcription or gene expression levels, fragments per kilobase of exon model per million mapped fragments (FPKM) values were used. To identify differentially expressed genes (DEGs), DESeq2 software and the Benjamini−Hochberg method were used (Love et al., 2014; Varet et al., 2016). The DEGs met the criteria |log_2_Fold Change|≥1 and false discovery rate (FDR) < 0.05. DEG enrichment was assessed based on comparison with Gene Ontology (GO) and KEGG data. The transcriptome data are available in the NCBI database (https://www.ncbi.nlm.nih.gov/) under BioProject PRJNA881405 and PRJNA934137. qRT−PCR (real-time fluorescence quantitative PCR) was used to confirm the gene expression results. As targets for analysis, 11 structural and regulatory genes related to the anthocyanin pathway were selected. Total RNA from green and red leaf tissues at three developmental stages was extracted and then employed to synthesize cDNA. The primer sequences employed are shown in **Table S3**, and *β*-actin was used as an internal reference gene. qRT−PCR was conducted on a LightCyclerR480 system (Roche, Switzerland) as described in a previous article (Chen et al., 2022a).

TFs were annotated with iTAK software and then subjected to BLAST searches with PlnTFDB and PlantTFDB. Based on the measured gene expression levels and metabolite contents, a network of TFs, structural genes, and metabolites was constructed. The selected genes and metabolites are listed in **Table S4**. The correlation network was constructed in the MetWare Cloud (https://cloud.metware.cn/) according to the following settings: the correlation analysis method was Pearson, the numerical conversion method was log2, the correlation analysis threshold was 0.8, and the *P* value threshold for the significant difference was 0.05. The network was visualized in a concentric circle diagram using Cytoscape software (v3.8.2) and was distributed using the degree algorithm. Phylogenetic trees of MYB and bHLH amino acid sequences were constructed and downloaded from the NCBI database. The phylogenetic tree was drawn with MEGA software (v6.0) using the neighbor-joining method. Correlation analysis of the transcriptome and metabolome data was carried out based on published methods (Yang et al., 2021). The genes and metabolites related to the flavonoid pathway (k00941) and anthocyanin pathway (k00942) are displayed.

Weighted gene coexpression network analysis (WGCNA) is used to find co-expressed gene modules and explore the association between gene networks and phenotypes (Langfelder & Horvath, 2008). The module is defined as a group of genes with similar expression profiles. If certain genes always show similar expression changes during a physiological process, it is reasonable to consider these genes to be functionally related; they can be defined as a module (Liu et al., 2020). First, the gene expression data are processed. The correlation coefficient between any two genes is calculated using the weighted correlation method. In the second step, a hierarchical clustering tree is constructed from the correlation coefficients between genes. Genes are classified according to their expression patterns, and genes with similar patterns are grouped into a module. In this way, tens of thousands of genes can be divided into dozens of modules according to their expression patterns. The corresponding modules are merged into the same module. The final merged modules are used for subsequent analyses. In the third step, the modules associated with the traits were identified. The correlation coefficients between gene modules and phenotypes were calculated using the Pearson correlation algorithm. The trait must be a numerical trait. In this study, WGCNA was used to analyze the correlation between the gene network and the total anthocyanin content. Bioinformatic analysis was performed using OECloud tools at https://cloud.oebiotech.cn according to the following settings: the power value was 30, the threshold value for standard deviation filtering was 0.5, and the threshold value for module merging was 0.25.

## 3 Results

### 3.1 Variations in phenotypes and biochemical analyses during leaf development

There are three distinct stages (R1, R2, and R3) of color transition in red leaves, and there are three corresponding developmental stages (G1, G2, and G3) in green leaves (**Figure 1A**). The green leaves remain green to the naked eye, regardless of the changes in red leaves. The chlorophyll a (**Figure 1B**), chlorophyll b (**Figure 1C**), total chlorophyll (**Figure 1D**), and carotenoid contents (**Figure 1E**) of the red leaves at different stages were much higher than those of the green leaves. However, the total anthocyanin content in the red leaves was higher than that in the green leaves in all three stages (**Figure 1F**). In the first stage, the anthocyanin content accounted for a high proportion of all pigments compared to the other two stages (**Figure 1G**). Moreover, the contents of flavonoids and polyphenols in red leaves at the first stage were considerably greater than those in green leaves. The total flavonoid content in R1 was 6.46 times that in G1 (**Figure 1H**), and the total polyphenol content in R1 was 8.25 times that in G1 (**Figure 1I**). DPPH and ABTS^+^ assays were performed to measure the total antioxidant ability. The DPPH and ABTS values in R1 were significantly higher than those in G1 (**Figures 1J, K**). It could be preliminarily inferred that polyphenols, flavonoids, and anthocyanins were the principal differentially abundant metabolites between red and green leaves.

### 3.2 Analysis of bioactive flavonoids in green and red leaves of *Populus × euramericana* ‘Zhonghuahongye’

**Figure 2:**
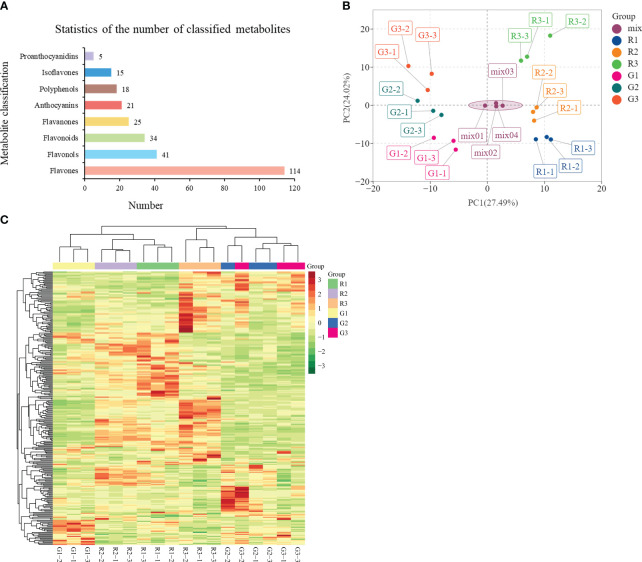
Analysis of flavonoid metabolites in green and red leaves. **(A)** Statistics of the number of classified metabolites. **(B)** Principal component analysis (PCA) of the samples from three developmental stages and quality control samples (mix). The x-axis represents the first principal component, and the y-axis represents the second principal component. **(C)** Cluster heatmap of all samples.

Flavonoid metabolites were analyzed in the three periods of leaf color change in red leaf material (R1, R2, and R3) and green leaf material (G1, G2, and G3). A total of 273 flavonoid metabolites were identified (**Table S5**), which were mainly divided into eight categories, among which flavones and flavonols were the most numerous (114 and 41, respectively), followed by flavonoids (34), flavanones (25), anthocyanins (21), polyphenols (18), isoflavones (15), and proanthocyanidins (5) (**Figure 2A**). PCA showed large differences in the metabolite composition in different developmental stages of red leaves and green leaves (**Figure 2B**). The PCA results showed a clear clustering of metabolic profiles based on leaf color and developmental period. The 1st, 2nd, and 3rd principal components accounted for 27.49%, 24.02%, and 12.18% of the total variability, respectively (**Figure S2**). In the OPLS-DA model, the Q^2^ values of pairwise comparisons exceeded 0.9 (**Figure S3**). The cluster diagram of all samples showed that the metabolites from R1, R2, and G1 had similar expression patterns. There were obvious differences between the R3 and the R1 and R2 stages. The metabolites of R2 and G2 had significant differences and were assigned to two branches of the cluster (**Figure 2C**). These results provide a basis for further analysis of metabolite differences in the samples.

The flavonoids in *Populus × euramericana* ‘Zhonghuahongye’ leaves were analyzed. In the flavonoid category, the bioactive ingredients isoquercitroside, liquiritin, and the rare narcissoside were identified. In the flavonol category, the valuable components quercetin, kaempferol, quercetin 3-*O*-rutinoside (rutin), kaempferol 7-*O*-rhamnoside, kaempferol 3-*O*-rutinoside (nicotiflorin), kumatakenin, dihydroquercetin (taxifolin), and isorhamnetin were found. In the flavone class, baicalein-7-*O*-glucuronide was identified. In the bioactive ingredient isoflavone class, daidzein, genistein, galycosin, and prunetin were identified. In the medicinal polyphenol class, catechin, L-epicatechin, epicatechin gallate (ECG), and theaflavin were identified. The procyanidin components A3, A1, A2, B2, and B3 were detected. Furthermore, in the anthocyanin class, cyanidin 3-*O*-galactoside, cyanidin 3,5-*O*-diglucoside, peonidin *O*-hexoside, cyanidin *O*-syringic acid, delphinidin 3-*O*-rutinoside, delphinidin *O*-malonyl-malonylhexoside, and peonidin 3-*O*-glucoside chloride were identified. These representative compounds have been proven to have antioxidant and anti-inflammatory properties *in vitro*, and most of them showed relatively high abundances in red leaves of the R1 and R2 stages (**Figure S4**). Other phytochemical components are listed in **Table S5** for further analysis.

### 3.3 Analysis of differentially abundant flavonoid metabolites between red and green leaves and their KEGG classification

**Figure 3:**
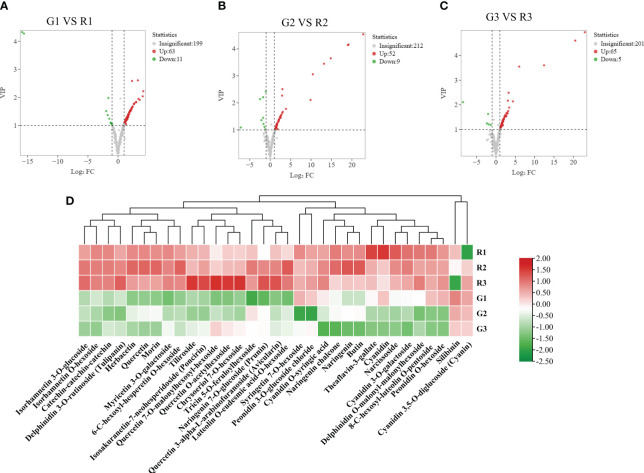
Differentially abundant metabolite screening. **(A)** Volcano plot of differentially abundant metabolite clustering for the G1 vs. R1 comparison. **(B)** Volcano plot of DEMs for the G2 vs. R2 comparison; **(C)** volcano plot of DEMs for the G3 vs. R3 comparison; **(D)** metabolites with significant differences in three developmental stages of poplar green (G1, G2, and G3) and red (R1, R2, and R3) leaves. The volcano plot map reflects the differences in metabolite expression levels in the two samples. The vertical coordinates represent the VIPs of the OPLS-DA model, and the horizontal coordinate values are the log2 (fold changes). Each point in the graph represents a detected metabolite. The legend of the volcano plot indicates the number of significant DEMs.

To reveal the accumulation patterns of flavonoids in the red and green leaves of *Populus × euramericana* ‘Zhonghuahongye’ poplar, pairwise comparisons of G1 vs. R1, G2 vs. R2, and G3 vs. R3 were conducted. Seventy-four significant DEMs were identified between G1 and R1 (63 increased and 11 decreased) (**Figure 3A**). In addition, 62 significant DEMs were identified in G2 vs. R2 (53 increased and 9 decreased) (**Figure 3B**), and 71 significant DEMs were identified in G3 vs. R3 (66 increased and 5 decreased) (**Figure 3C**). Most flavonoid metabolites accumulate in large quantities in red leaves. Venn diagram analysis showed that 33 significant DEMs were shared by the G1 vs. R1, G2 vs. R2, and G3 vs. R3 comparison groups (**Figure S5**). These flavonoids were considered the ‘core metabolite group’ in poplar leaves (**Table S6**). The hierarchical clustering analysis showed that most of the ‘core metabolites’ were upregulated in the red leaves compared with the green leaves, other than silibinin and cyanidin 3,5-*O*-diglucoside (**Figure 3D**). This implies that the 31 ‘core metabolites’ of flavonoids (7 flavones, 6 flavonols, 5 flavonoids, 4 flavanones, 7 anthocyanins, and 2 polyphenols) were enriched in red poplar leaves.

The KEGG classification results showed that the significant DEMs of G1 vs. R1 were mainly involved in the isoflavonoid biosynthesis, flavonoid biosynthesis, flavone and flavonol biosynthesis, and anthocyanin biosynthesis pathways (**Figure S6A**). The significant DEMs of G2 vs. R2 were mainly enriched in flavonoid biosynthesis, anthocyanin biosynthesis, and isoflavonoid biosynthesis (**Figure S6B**). The significant DEMs of G3 vs. R3 were mainly related to flavonoid biosynthesis, anthocyanin biosynthesis, flavone and flavonol biosynthesis, and isoflavonoid biosynthesis, with the *P* value of anthocyanin biosynthesis being the smallest among the four pathways (**Figure S6C**). The KEGG enrichment classification histograms are shown in **Figure S4D**. The results indicated that leaf color presentation may be influenced by anthocyanin biosynthesis and flavonoid biosynthesis. Thereafter, the significant DEMs of the anthocyanin class in G1 vs. R1, G2 vs. R2, and G3 vs. R3 were analyzed (**Table 1**). Nine anthocyanins, including peonidin *O*-hexoside, delphinidin *O*-malonyl-malonylhexoside, cyanidin *O*-syringic acid, malvidin 3-*O*-galactoside, malvidin 3-*O*-glucoside, delphinidin 3-*O*-rutinoside, cyanidin, cyanidin 3-*O*-galactoside, and peonidin 3-*O*-glucoside chloride, were upregulated in R1 compared with G1. In addition to the anthocyanins mentioned above, cyanidin 3-*O*-glucoside showed upregulated expression in R2 compared with G2, and cyanidin 3-*O*-glucoside, delphinidin, and malvidin 3-acetyl-5-diglucoside were significantly upregulated in R3 compared with G3. Notably, the majority of anthocyanins, especially peonidin *O*-hexoside, malvidin 3-*O*-galactoside, peonidin 3-*O*-glucoside chloride, and cyanidin 3-*O*-glucoside, were more abundant in all three developmental stages of red leaves than in green leaves. Thus, these results suggest that the differences in the anthocyanin compositions are responsible for the differences in the leaves of *Populus* × *euramericana* ‘Zhonghuahongye’.

**Table 1:** Anthocyanin accumulation in different periods.

Anthocyanins	G1 vs R1^a^		G2 vs R2^b^		G3 vs R3^c^	
	Log_2_FC^d^	KEGG^e^	Log_2_FC	KEGG	Log_2_FC	KEGG
Peonidin O-hexoside	2.63	Nd	19.19	Nd	20.49	Nd
Rosinidin O-hexoside	-1.45	Nd	-1.73	Nd	NS	NS
Delphinidin O-malonyl-malonylhexoside	1.42	Nd	2.43	Nd	2.22	Nd
Cyanidin O-syringic acid	1.09	Nd	2.41	Nd	4.38	Nd
Malvidin 3-O-galactoside	3.60	Nd	14.82	Nd	NS	NS
Malvidin 3-O-glucoside	4.09	ko00942	1.47	ko00942	NS	NS
Cyanidin 3,5-O-diglucoside	-15.61	ko00942	-1.07	ko00942	2.39	ko00942
Malvidin 3,5-diglucoside	-1.72	Nd	NS	NS	NS	NS
Delphinidin 3-O-rutinoside	1.22	ko00942	2.15	ko00942	1.85	ko00942
Cyanidin	2.71	ko00941 ko00942	2.72	ko00941 ko00942	3.01	ko00941 ko00942
Cyanidin 3-O-galactoside	2.20	Nd	3.07	Nd	3.40	Nd
Peonidin 3-O-glucoside chloride	2.64	Nd	19.02	Nd	6.00	Nd
Cyanidin 3-O-glucoside	NS	NS	22.77	ko00942	22.99	ko00942
Delphinidin	NS	NS	NS	NS	2.20	ko00941 ko00942
Malvidin 3-acetyl-5-diglucoside	NS	NS	NS	NS	1.96	Nd

^a^ G1 vs R1 refers to the content of metabolites in the R1 group of red leaves compared with that in the G1 group of green leaves. The G1 is the control group in this comparison. ^b^ G2 vs R2 refers to the content of metabolites in the R2 group of red leaves compared with that in the G2 group of green leaves. The G2 is the control group in this comparison. ^c^ G3 vs R3 refers to the content of metabolites in the R3 group of red leaves compared with that in the G3 group of green leaves. The G3 is the control group in this comparison. ^d^ Log_2_FC is Log_2_ (fold change). ^e^ KEGG refers to metabolites are annotated to the pathway of KEGG database. ‘ND’ indicates this metabolite was not found in the KEGG enrichment pathway. ‘NS’ indicates that there is no significant difference in comparison group.

### 3.4 Analysis of the transcriptome and DEGs of green leaves and red leaves

**Figure 4:**
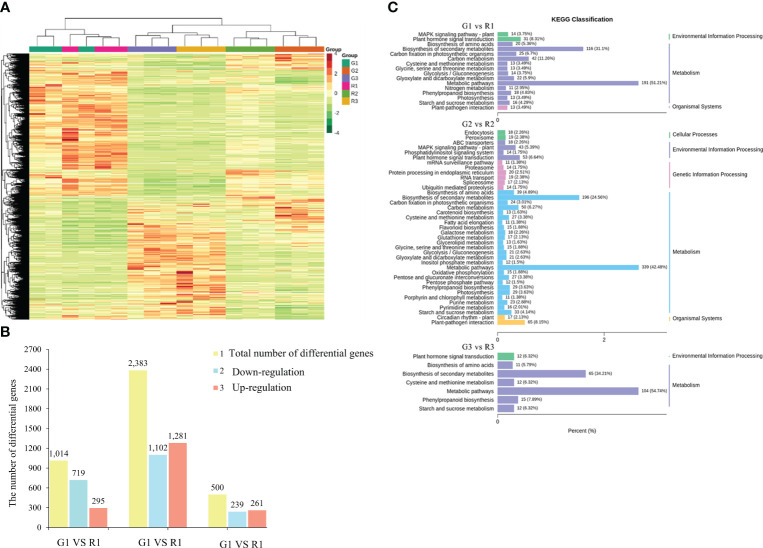
Differential gene expression. **(A)** Heatmap of differentially expressed gene clusters. **(B)** The number of genes with differences in the comparison of red leaves and green leaves. **(C)** Column chart of KEGG classification of DEGs between red and green leaves. The abscissa represents the ratio of annotated genes to the total number of genes annotated, and the ordinate represents the name of the KEGG pathway. The labels on the right side of the graph represent the category to which the KEGG pathway belongs.

RNA-Seq was used for genome-wide gene expression profiling in the red leaves and green leaves of *Populus* × *euramericana* ‘Zhonghuahongye’. A total of 134.1 Gb of clean data was generated, with an average GC content of 44.10% (**Table S7**). The matching rate of each sample was 81.12-85.19% (**Table S8**). A total of 34,200 genes were identified, among which 15,019 DEGs were identified by taking green leaves as the control group and red leaves as the experimental group. Then, hierarchical clustering of DEGs across all samples revealed the transcriptomic profiles of normal red leaf and green leaf tissues during leaf development in *Populus* × *Euramerica* ‘Zhonghuahongye’ (**Figure 4A**). The DEGs of normal red leaves and green leaves (G1 vs. R1, G2 vs. R2, G3 vs. R3) in different developmental stages were analyzed. A total of 1014 DEGs were obtained from the G1 vs. R1 group, among which 295 genes (29.09%) showed upregulated expression, and 719 genes (70.91%) showed downregulated expression, indicating that the difference in leaf color was mainly regulated by genes with downregulated expression in the first stage (**Figure 4B**). However, the highest number of DEGs (2383) was obtained in G2 vs. R2, and the lowest number of 500 DEGs was obtained in G3 vs. R3. The genes with upregulated expression accounted for 52.20% of all DEGs in the second stage and 53.76% in the third stage. Next, a Venn diagram was generated to show the distribution of DEGs in different developmental stages (**Figure S7**). There were 609 unique DEGs in the G1 vs. R1 group, 1875 unique DEGs in the G2 vs. R2 group, and 217 DEGs in the G3 vs. R3 group. Seventy-six common DEGs were screened in these three comparison groups. Taken together, these results suggest that differential gene expression in the first and second stages plays a crucial role in leaf color differences.

To understand the functions of the DEGs, GO and KEGG enrichment analyses were performed. The GO enrichment results showed that the DEGs of the G1 vs. R1 group were significantly enriched in biological processes such as photosynthesis, carbohydrate biosynthesis, hormone metabolism, apoplast, and other biological processes (**Figure 4C**). The DEGs of G2 vs. R2 were enriched in the carbohydrate biosynthesis process, cell cycle process, cytoskeleton, and other cellular components, as well as in UDP glycosyltransferase activity and other molecular functions. The DEGs of G3 vs. R3 were enriched in pigment metabolism, isoprenoid metabolism, terpene metabolism, and UDP glycosyltransferase activity. The KEGG enrichment results showed that the 1016 DEGs of the G1 vs. R1 group were annotated into 116 metabolic pathways. Among these pathways, the carbon metabolism (42 DEGs, 11.26%), plant hormone signal transduction (31 DEGs, 8.31%), and phenylpropanoid biosynthesis (18 DEGs, 4.83%) pathways were significantly enriched (**Figure 4C**). The 2383 DEGs of G2 vs. R2 were enriched in 122 metabolic pathways. In addition to the abovementioned pathways identified in G1 vs. R1, the starch and sucrose metabolism (33 DEGs, 4.14%), glycolysis-related metabolism, ABC transporters (18 DEGs, 2.26%), glutathione metabolism (17 DEGs, 2.13%), and flavonoid biosynthesis (15 DEGs, 1.88%) pathways were annotated in this group (**Figure 4C**). The DEGs of G3 vs. R3 were enriched in 95 metabolic pathways. In general, the numbers of KEGG pathways and their types in G2 vs. R2 were significantly higher than those in the other two groups, indicating that more complex biological activities were carried out in leaves in the second stage.

### 3.5 Construction of the gene coexpression network during leaf coloration based on WGCNA

To explore the genetic regulatory networks of anthocyanin biosynthesis in green and red leaves, WGCNA was conducted based on all genes and the total anthocyanin content. All the filtered genes of green and red leaves were analyzed by WGCNA. The modules were classified according to the hierarchical clustering tree, and a total of 12 merged dynamic modules were generated (**Figure S8A**). The genes in a module have similar expression profiles. The midnightblue module contained the most genes (4826), while the bisque4 module contained the fewest (51) (**Figure S8B**). In the midnightblue module, the Pearson correlation coefficient of genes and total anthocyanins in R1 was 0.67, which was higher than that in G1 (0.48). The red color of R1 and G1 in the heatmap shows that the high expression of genes and high total anthocyanin content in the first stage was strongly and positively correlated. The redder the color was, the stronger the correlation. The Pearson correlation coefficient in R3 was -0.45. The blue color of R3 in the heatmap indicates that those genes in the third stage were negatively correlated with the total anthocyanin content. The results of anthocyanin determination indicate that the content was higher in red leaves than in green leaves at each time point. The changes in the Pearson correlation coefficient among the midnightblue module were consistent with the anthocyanin content. The Eigengene expression histogram shows the distribution of gene expression variables in each sample in the module (**Figure S8C**). The Eigengene expression histogram is a different method for visualization of the obtained results. Thus, the results suggested that the genes in the midnightblue module might be associated with the anthocyanin content. Therefore, the midnightblue module was selected for further analysis.

The GO enrichment analysis of genes revealed high enrichment of BP (biological process) terms, CC (cellular component) terms, and MF (molecular function) terms. The genes in the midnightblue modules were highly enriched in BP terms (**Figure S9A**; **Table S9**), including signaling (GO:0023052), response to hormone (GO:0009725) and response to abscisic acid (GO:0009737), and CC terms, including nucleus (GO:0005634), endomembrane system (GO:0012505), and vacuole (GO:0005773). The top 20 enriched KEGG pathways were mainly related to starch and sucrose metabolism, plant hormone signal transduction, glycolysis/gluconeogenesis, and the MAPK signaling pathway (**Figure S9B**; **Table S10**). Phenylpropanoid biosynthesis and photosynthesis-related pathways were also enriched. In the starch and sucrose metabolism pathways, the *HXK* (hexokinase) gene (*POPTR_001G190400v3*) and beta-glucosidase genes (*POPTR_002G114000v3* and *POPTR_004G019800v3*) were identified. In the plant hormone signal transduction pathways, auxin-responsive *GH3* genes (*POPTR_001G298300v3* and *POPTR_003G161300v3*) and *SAUR* genes (*POPTR_002G000600v3* and *POPTR_002G024500v3*) were annotated. The *SnRK2* (serine/threonine-protein kinase SRK2) gene (POPTR_002G099700v3) is related to the ABA signaling pathway. Flavonoid and anthocyanin biosynthesis genes, including four *HCT* genes (*POPTR_005G028200v3*, *POPTR_005G052200v3*, *POPTR_008G034100v3*, *POPTR_019G001400v3*), *CHS* (POPTR_012G138800v3), and *ANS* (*POPTR_016G117100v3*), were differentially expressed. The above genes might play important roles in the higher anthocyanin content in red leaves.

### 3.6 TFs related to flavonoid and anthocyanin dynamics in *Populus × euramericana* ‘Zhonghuahongye’ leaves

**Figure 5:**
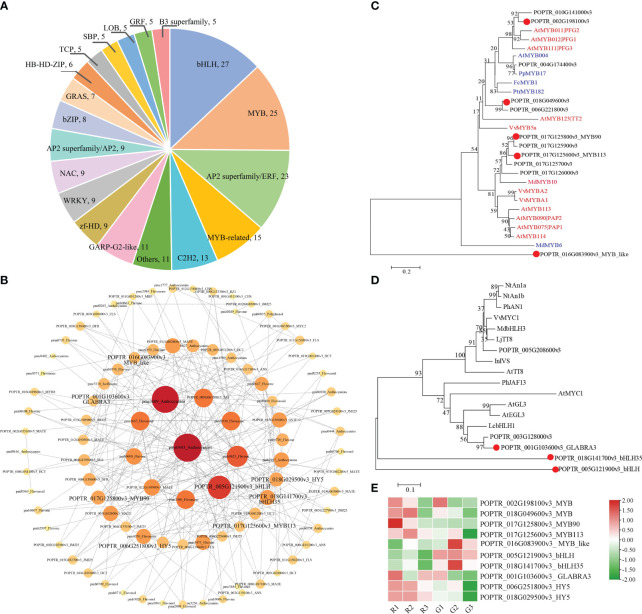
Identification of TFs involved in the biosynthesis of flavonoids and anthocyanins **(A)** Classifications and proportions of TFs. Each section shows the names and numbers of TF families. **(B)** Network among structural genes, transporter genes, TFs, and metabolites involved in the flavonoid and anthocyanin pathways. **(C)** Phylogeny of putative MYBs in *Populus* × *euramericana* ‘Zhonghuahongye’ and other known MYBs related to flavonol, flavonoids, proanthocyanidins, and anthocyanins in other species. In these phylogenetic relationships, the red ID represents a TF that promotes synthesis, and the blue ID is a TF that inhibits synthesis. **(D)** Phylogeny of putative bHLHs and other known bHLHs related to anthocyanins in other species. The GenBank IDs and annotations of MYB and bHLH TFs are listed in **Table S12**. The distance scale represents the unit length of the difference value between sequences. The bootstrap value is used to evaluate the credibility of the branch. **(E)** The expression patterns of 10 genes encoding TFs. The FPKM values were normalized by the Z score method in TBtools software.

TFs are important regulators in the regulatory network of flavonoid biosynthesis. A total of 278 DEGs encoding TFs were identified in the G1 vs. R1, G2 vs. R2, and G3 vs. R3 comparisons (**Table S11**). The top 20 TFs were identified, and the top 5 categories were bHLH (27), MYB (25), AP2 superfamily/ERF (23), MYB related (25), and C2H2 (13) (**Figure 5A**). The network results showed that MYB TFs, bHLH TFs, and bZIP (basic leucine zipper) family HY5 (elongated hypocotyl 5) TFs may mediate flavonoid and anthocyanin metabolism (**Figure 5B**). The phylogenetic analysis results showed that the MYB TF (POPTR_002G198100v3) clustered with the flavonol induction TFs AtPFG1 and AtPFG2. POPTR_017G125800v3_MYB90 and POPTR_017G125600v3_MYB113 clustered with anthocyanin-promoting MYB TFs (**Figure 5C**). The phylogenetic results for bHLH TFs showed that three candidate bHLHs presented high homology with the known bHLH sequence regulating flavonoids (**Figure 5D**). In particular, the translated POPTR_001G103600v3_GLABRA3 amino acid sequence was similar to the LcbHLH3 sequence of *Litchi chinensis* Sonn., which interacts with LcMYB1 to enhance anthocyanin accumulation (Lai et al., 2016). Expression pattern analysis showed that five MYB TFs, three bHLH TFs, and two HY5 TFs were significantly differentially expressed (**Figure 5E**). The putative flavonol accumulation-related TFs POPTR_002G198100v3_MYB and POPTR_002G198100v3_MYB showed higher expression in the R1, R2, and G1 stages, consistent with the accumulation pattern of flavonoid metabolites. The assumed anthocyanin synthesis promoters POPTR_017G125800v3_MYB90 and POPTR_017G125700v3_MYB113 showed upregulated expression in the R1 and R2 stages. In contrast, POPTR_016G083900v3_MYB_like showed downregulated expression in the R stage, which might have inhibited flavonoid synthesis. The bHLH TF POPTR_ 001G103600v3_ GLABRA3 showed upregulated expression, especially in the R1 stage. In addition, two HY5 TFs showed upregulated expression in the R1 and R2 stages of red leaves compared with green leaves (**Figure 5E**). These MYB, bHLH, and HY5 TFs played key roles in flavonoid and anthocyanin synthesis in *Populus* × *euramericana* ‘Zhonghuahongye’.

### 3.7 Relationships between flavonoid biosynthesis and metabolites

The correlation between metabolites and RNA-seq data was analyzed. A 9-quadrant diagram based on correlation analysis showed that the metabolite and gene expression patterns were consistent in the third and seventh quadrants (**Figure S10**). Among these, 20 genes associated with 9 metabolites were enriched in the flavonoid and anthocyanidin pathways. Two *CHI* genes and 4,2’,4’,6’-tetrahydroxy chalcone were identified as related. Four HCT genes, two *CHI* genes, an *ANR* gene, and dihydromyricetin were identified as related. Three *HCT* genes, four *FLS* (flavonol synthase) genes, an *ANS* gene, and naringenin 7-*O*-glucoside were identified as positively related. In addition, an *HCT* gene and delphinidin were identified as related. The *HCT* gene *CYP75B1* and luteolin were identified as related (**Figure S11**). To further confirm the reliability of the RNA-Seq results, the 11 candidate genes mentioned above were selected for verification. The RT−qPCR results showed that these gene expression patterns were consistent with the RNA-Seq results (**Figure S12**).

**Figure 6:**
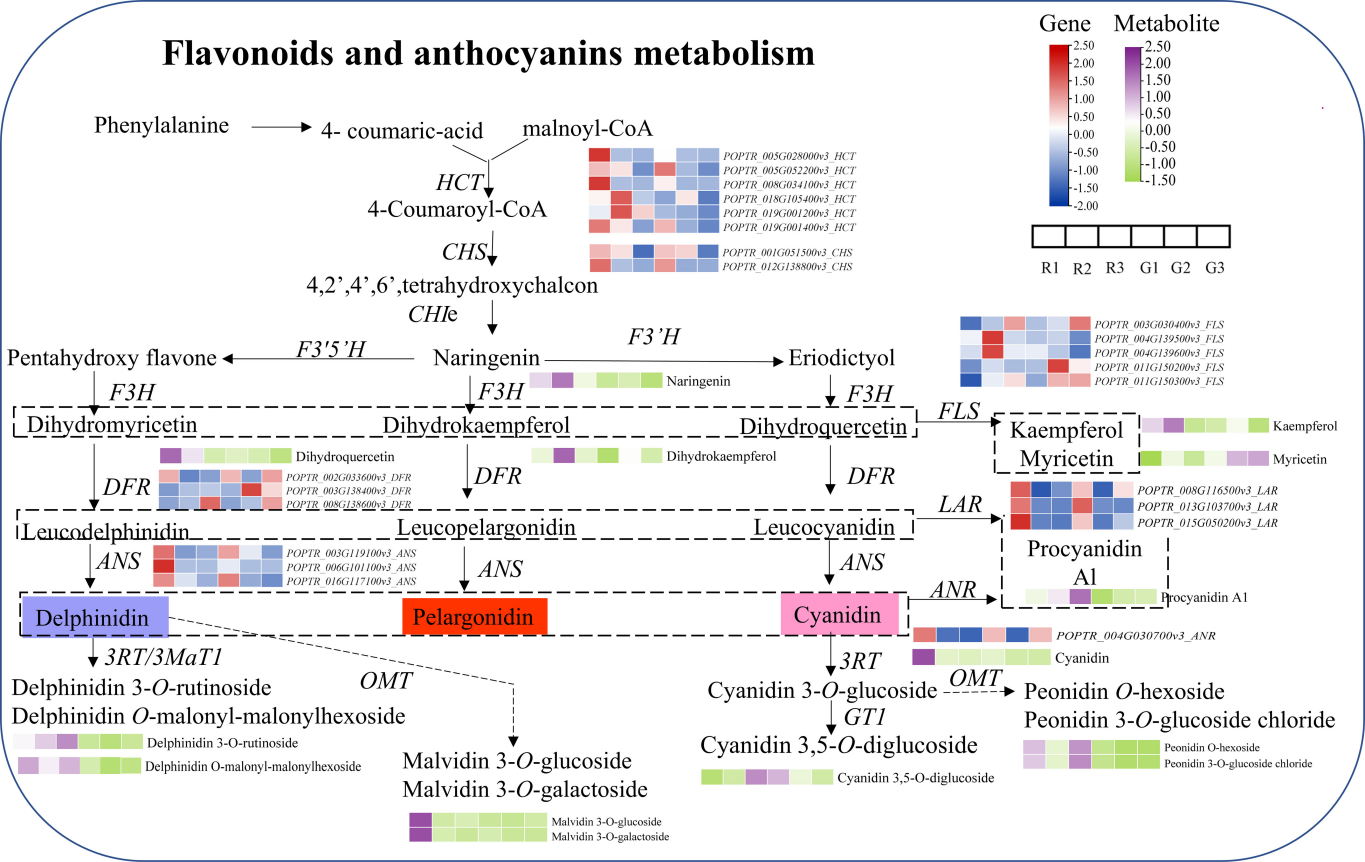
Combined analysis of metabolites and genes involved in flavonoid and anthocyanin biosynthesis. The purple and yellow−green heatmaps indicate the accumulation of metabolites. Blue and red heatmaps show gene expression. HCT, Hydroxycinnamoyl CoA: shikimate hydroxycinnamoyl transferase; PAL, phenylalanine ammonia-lyase; C4H, cinnamic acid 4-hydroxylase; 4CL, 4-coumaric acid: CoA-ligase; CHS, chalcone synthase; CHI, chalcone isomerase; F3H, flavanone 3-hydroxylase; F3’,5’H, flavonoid 3’,5’-hydroxylase) or F3’H, flavonoid 3’-hydroxylase; DFR, dihydroflavonol 4-reductase; ANS, anthocyanidin synthase; FLS, flavonoid synthase; LAR, Leucoanthocyantin reductase; ANR, Anthocyanidin reductase; RT, glycosyltransferase; 3MaT1, anthocyanin 3-*O*-glucoside-6’’-*O*-malonyltransferase; and OMT, *O*-methyltransferase.

The combination analysis indicated that the expression of genes related to flavonoid synthesis in red leaves was higher than that in green leaves in the same developmental stage, including the *HCT* (shikimate O-hydroxycinnamoyl-transferase gene), *CHS*, *DFR*, and *ANS* genes (**Figure 6**). Under the action of DFR, dihydromyricetin and dihydroquercetin are transformed into leucoanthocyanin. Leucoanthocyanins are generated as anthocyanins under the action of ANS, so anthocyanins (such as delphinidin and cyanidin) accumulate in young red leaves (R1). With the development of red leaves, the *CHS*, *DFR*, and *ANS* genes were continuously downregulated in the subsequent developmental stages (R2 and R3). In contrast, *FLS* showed continuous upregulation, which promoted the synthesis of flavonol. The high expression of *LAR* (leucoanthocyanin reductase gene) and *ANR* (anthocyanidin reductase gene) led to the conversion of leucoanthocyanin and anthocyanin to proanthocyanidin A1. The substrate of anthocyanin synthesis in red leaves was consumed, which may be an important reason for the color change in red leaves. Similarly, the high expression of *FLS* in green leaves resulted in the inability to synthesize anthocyanins in green leaves. The continuous downregulation of naringin, dihydromyricetin, cyanidin, and malvidin 3-*O*-glucoside might be responsible for the color change in red leaves of *Populus* × *euramericana* ‘Zhonghuahongye’ from the R1 to R3 stages. However, in the green leaf developmental period, the contents of cyanidin 3,5-*O*-diglucoside, peonidin O-hexoside, and malvidin 3-*O*-galactoside decreased significantly. Compared with green leaves, delphinidin 3-*O*-rutinoside, malvidin 3-*O*-glucoside, malvidin 3-*O*-galactoside, and peonidin *O*-hexoside showed the most significant increases in red leaves during the three stages.

## 4 Discussion

### 4.1 Insight into the potential utility of *Populus × euramericana* ‘Zhonghuahongye’

*Populus* extract has high therapeutic potential because of its antioxidant, anti-inflammatory, hepatoprotective, antitumor, and antimicrobial properties. More than 100 compounds have been isolated from *Populus nigra* extract, including phenolic compounds, terpenoids, flavonoids, flavanone, caffeic and p-coumaric acids, and nearly 50 molecules from the essential oil of its buds (Tebbi & Debbache-Benaida, 2022). To date, several studies have focused on the chemical composition of *Populus* red leaves (Tian et al., 2021; Chen et al., 2023), whereas no further analysis has been conducted on the difference in the composition between red and green leaves throughout the lifecycle. In this study, we profiled the flavonoid compositions of the red and green leaves of *Populus × euramericana* ‘Zhonghuahongye’ at three stages. A total of 273 flavonoid metabolites were identified in the three stages of leaf development. Among these metabolites, flavones, and flavonols accounted for the largest proportion, and 21 kinds of anthocyanins were identified. In the first stage, compared to green leaves, red leaves exhibited higher levels of total flavonoids, total phenols, and antioxidant activity. The metabolite results indicate that at each stage of red leaf development, the contents of most flavonoid metabolites in red leaves were higher than those in green leaves. Sixty-three significant DEMs were upregulated, while eleven DEMs were downregulated in R1 compared with G1. In addition, fifty-two significant DEMs were upregulated, while nine DEMs were downregulated, in R2 compared with G2. In addition, sixty-six significant DEMs were upregulated, while five DEMs were downregulated, in R3 compared with G3. In the various developmental stages of *Populus × euramericana* ‘Zhonghuahongye’, most of the 31 core flavonoid metabolites exhibited relatively high contents in red leaves at the three developmental stages, including 7 flavonoids, 6 flavonoids, 5 flavonoids, 4 flavonoids, 7 anthocyanins, and 2 polyphenols. In Tian’s study, only 210 flavonoid metabolites were identified in two red-leaf poplar cultivars (*Populus* sp. bright red leaf and completely red leaf varieties) based on a widely targeted metabolomic analysis on the HPLC−ESI−MS/MS platform (Tian et al., 2021). In this study, more metabolites were detected, which may be due to the selection of multiple leaf developmental stages. Compared with the green leaf variety of *Populus* sp. Linn. ‘2025’ collected in September 2018, 48 and 8 flavonoids were more and less abundant, respectively, in *Populus deltoides* varieties with bright red leaves, whereas 51 and 9 flavonoids were more and less abundant, respectively, in *Populus deltoides* varieties with completely red leaves (Tian et al., 2021). In Chen’s study, at three stages of leaf development (R1, R2, and R3) of *Populus × euramericana* ‘Zhonghuahongye’, 81 differentially abundant metabolites were detected in the R1 vs. R3 comparison, 45 were detected in the R1 vs. R2 comparison, and 75 were detected in the R2 vs. R3 comparison. Ten metabolites showed significant differences in all comparisons, which were mostly flavonoid metabolites (Chen et al., 2023). Unlike previous reports, our study observed leaves from the tender stage to the mature stage of *Populus × euramericana* ‘Zhonghuahongye’. More types of metabolites were identified in the metabolome, and more DEMs between the two leaves were identified. Among the 31 metabolites, naringenin, Butin, myricetin 3-O-galactoside, quercetin, cyanidin, and other compounds were highly expressed in red leaves. The antioxidant activity of the red leaf extract was higher due to the presence of flavonoids, polyphenols, and anthocyanins. Poplar leaves have been used as feed for ruminants, especially for goats and buffaloes (Ayers et al., 1996; Kumar et al., 2022). Thus, *Populus × euramericana* ‘Zhonghuahongye’, which is rich in flavonoids, has a higher feeding value than common green leaves. There is potential to develop the use of bioactive compounds from poplar red leaves.

### 4.2 Flavonoid metabolism is responsible for the difference between red and green leaves of *Populus × euramericana* ‘Zhonghuahongye’

Flavonoids have been studied in depth for more than a century (Koes et al., 2005). It is critical to further clarify the molecular basis of flavonoid biosynthesis (Chen et al., 2022b; Kundan et al., 2022). Poplar is recognized as a model perennial woody plant. Thus, this study aimed to elucidate the process of flavonoid formation in red leaves and green leaves of the *Populus* × *euramericana* ‘Zhonghuahongye’ variety. First, 4-coumaric acid and malonyl-CoA were found to be regulated by high expression of HCT genes in the R1 and R2 stages, followed by the CHS gene. The relative content of naringenin in R1 and R2 was approximately three times that in the G1 and G2 stages. Then, dihydroquercetin and kaempferol also exhibited increased levels in the R1 and R2 stages. Dihydrokaempferol was only upregulated in the R2 and G2 stages, while myricetin was abundant in the green leaves of the G2 and G3 stages. Naringenin, dihydroquercetin, kaempferol, dihydrokaempferol, and procyanidin A1 accumulated at higher levels in red leaves than in green leaves. In fig (*Ficus carica* L.) fruit, a very significant accumulation of the colorless flavonoids procyanidin B1, luteolin-3,7-di-*O*-glucoside, epicatechin, and quercetin-3-*O*-rhamnoside was observed in the mature “Purple Peel” compared to “Green Peel” (Wang et al., 2017). In *Actinidia arguta*, seven flavonoid compounds were closely associated with the pigmentation of red- and green-fleshed cultivars (Yu et al., 2020). The flavonoid profiles visualized by hierarchical cluster analysis indicated that *Populus* has a different flavonoid composition from *Ficus carica* L. and *Actinidia arguta*. Moreover, the flavonoid composition and contents vary among poplar species and clones (Donaldson et al., 2006; Pobłocka-Olech et al., 2021; Mazurek et al., 2022). Thus, we propose that red poplar varieties may be useful as models for studying flavonoids in trees.

Anthocyanins are responsible for coloration in most leaves, flowers, fruits, and seeds. This important feature has long attracted breeders and consumers. In chokecherry (*Padus virginiana*), the accumulation of malvidin 3-*O*-glucoside (violet) and pelargonidin 3-*O*-glucoside (orange−red) significantly correlated with the leaf color change from green to purple−red (Li et al., 2021). Tian et al. examined the following 8 anthocyanins, which did not exist in green leaves of L2025, in two red poplar varieties: cyanidin 3-*O*-glucoside, cyanidin 3-*O*-rutinoside, cyanidin 3,5-*O*-diglucoside, malvidin 3-*O*-galactoside, malvidin 3-*O*-glucoside, pelargonin, delphinidin 3-*O*-glucoside, and delphinidin 3,5-*O*-glucoside (Tian et al., 2021). However, in this study, the 8 anthocyanins had significant discrepancies in green leaves and red leaves among the three stages: cyanidin, cyanidin 3-*O*-galactoside, cyanidin *O*-syringic acid, cyanidin 3,5-*O*-diglucoside, delphinidin 3-*O*-rutinoside, delphinidin *O*-malonyl-malonylhexoside, peonidin *O*-hexoside, and peonidin 3-*O*-glucoside chloride. Interestingly, more peonidin anthocyanins associated with the leaf color difference were detected. In addition, while there was a difference in pelargonin abundance, it was not significant. In addition, centaverin 3-*O*-glucoside exhibited the highest expression in the three stages of red leaves. Therefore, these main anthocyanin compounds are responsible for the variance in leaf color of *Populus* × *euramericana* ‘Zhonghuahongye’.

### 4.3 A possible transcriptional activation network affects flavonoid and anthocyanin synthesis in *Populus × euramericana* ‘Zhonghuahongye’

*Populus*, as model plants, are considered excellent materials for studying flavonoid synthesis in woody plants. The biosynthesis of flavonoids/anthocyanin metabolites is also regulated by MYB and bHLH TFs (Yan et al., 2021). According to our data, five MYB TFs (POPTR_002G198100v3_MYB, POPTR_018G049600v3_MYB, MYB_like, MYB90, and MYB113) and three bHLH TFs (GLABRA3, bHLH, and bHLH35) may be key TFs that regulate the contents of flavonoids and anthocyanins in *Populus* × *euramericana* ‘Zhonghuahongye’. In previous studies, *PtrMYB117* gene overexpression led to the accumulation of anthocyanins, proanthocyanidins, and flavonols through the upregulation of the flavonoid 3’,5’-hydroxylase gene (Ma et al., 2021). Obvious upregulation of the R2R3-MYB gene was observed in red poplar varieties with bright red leaves and completely red leaves (Tian et al., 2021). The R3 domain may contain the motif [D/E]Lx2[R/K]x3Lx6Lx3R, which is responsible for the interaction with an R-like bHLH protein. In addition, coexpression of the *PalbHLH1* and *PalMYB90* genes in *Populus alba* increased flavonoid levels to strengthen pathogen resistance (Bai et al., 2019).

The bZIP TF HY5 plays a critical role in controlling flavonoid and anthocyanin accumulation in response to light (Shin et al., 2007; Zhang et al., 2011; Xian et al., 2023). Overexpression of *CtHY5* initially promoted *CtCHS1* expression and flavonoid content in *Carthamus tinctorius* L. protoplasts (Xian et al., 2023). Previous studies have also emphasized that three TFs (HY5, HYH, and TTG2) may directly participate in the regulation of anthocyanin synthesis in two varieties, ‘Quanhong’ and ‘Xuanhong’ (Chen et al., 2021). The promotion of anthocyanin accumulation by FvbHLH9 depends on the HY5-bHLH heterodimer in *Fragaria ananassa* Duch. (Li et al., 2020). This study showed that two HY5 TFs (POPTR_006G251800v3_HY5 and POPTR_018G029500v3_HY5) were highly expressed in young, fresh red leaves, and the expression level gradually decreased as the leaves turned red−green. Therefore, we speculate that the high expression of the HY5 gene promotes the synthesis of flavonoids and anthocyanins in R1 (when the leaves are bright red), resulting in red leaves in the R2 stage. With the development of leaves, the downregulation of the HY5 gene may reduce flavonoid and anthocyanin synthesis, resulting in red−green leaves. CHS, as a key gene in the biosynthesis pathway of flavonoids, was upregulated in the R1 and R2 stages. ANS gene products transformed the accumulated metabolites into key anthocyanins (delphinidin and cyanidin) in the young red leaves of *Populus* × *euramericana* ‘Zhonghuahongye’ compared with green leaves. This paper shows that the high activity of TFs (MYB-bHLH-HY5) and structural genes (*CHS* and *ANS*) triggered the early accumulation of molecules of the flavonoid and anthocyanin biosynthesis pathways in young, bright red leaves.

## 5 Conclusion

In this study, we focused on the diversity of flavonoids in the red and green leaves of *Populus* × *euramericana* ‘Zhonghuahongye’ in three developmental stages, combined with phenotypic observations, physiological determinations, and gene expression profiles. Young red leaves have higher anthocyanin, total flavonoid and polyphenol contents, and antioxidant ability. A total of 273 flavonoids with various modifications were detected using widely targeted metabolomics. Most of these flavonoids exhibited higher levels of accumulation in the young leaves of poplars compared with the green leaves in the same stage. In particular, peonidin *O*-hexoside, malvidin 3-*O*-galactoside, peonidin 3-*O*-glucoside chloride, cyanidin 3-*O*-glucoside chloride, and cyanidin 3-*O*-glucoside might be responsible for the differences between green and red leaves. RNA-seq analysis showed that the downregulated expression of the flavonoid biosynthesis genes *CHS*, *DFR*, and *ANS* in poplar resulted in decreased red color in red leaves. The profile also revealed differential expression of several flavonoid and anthocyanin regulators, five MYB, three bHLH, and two HY5 genes. These findings provide new insights into the utilization of bioactive flavonoids in red poplar leaves, as well as the existence of green leaves from the perspective of gene transcription and flavonoid metabolism.

## Data availability statement

The datasets presented in this study can be found in online repositories. The names of the repository/repositories and accession number(s) can be found below: BioProject PRJNA881405 and PRJNA934137.

## Author contributions

YY: Data curation, Visualization, Writing – original draft, Writing – review & editing. MC: Conceptualization, Data curation, Formal analysis, Investigation, Methodology, Software, Validation, Writing – original draft, Writing – review & editing. HL: Investigation, Methodology, Validation, Writing – original draft. WZ: Data curation, Investigation, Methodology, Writing – original draft. HZ: Investigation, Validation, Writing – original draft. XN: Writing – review & editing. ZZ: Investigation, Validation, Writing – original draft. XH: Methodology, Validation, Writing – original draft. JZ: Investigation, Project administration, Resources, Supervision, Validation, Writing – review & editing.

## Supplementary material

Supplementary Figure 1 The sampling operations.

Supplementary Figure 2 PCA results show a clear clustering of metabolic profiles based on leaf color and developmental period.

Supplementary Figure 3 Metabolomic data were analyzed based on the OPLS-DA model.

Supplementary Figure 4 Representative bioactive flavonoids. The heatmap was generated based on the relative contents of metabolites. Red represents high abundance in these samples, and green represents low abundance.

Supplementary Figure 5 Venn diagram showing the number of DEMs identified in the pairwise comparisons and the common DEMs.

Supplementary Figure 6 KEGG enrichment bubble plots for DEMs in G1 vs. R1, G2 vs. R2, and G3 vs. R3.

Supplementary Figure 7 Venn diagrams of differentially expressed genes in red leaves and green leaves.

Supplementary Figure 8 WGCNA of filter genes and the total anthocyanin content.

Supplementary Figure 9 The GO and KEGG enrichment of genes in the midnightblue modules.

Supplementary Figure 10 A 9-quadrant diagram based on correlation analysis of metabolites and genes in the G1 vs. R1, G2 vs. R2, and G3 vs. R3 comparisons.

Supplementary Figure 11 The 20 genes associated with 9 metabolites were enriched in the flavonoid and anthocyanin pathways.

Supplementary Figure 12 qRT−PCR results of 11 candidate genes. β-Actin was used as an internal reference gene.
