# Supplementary figures and images for: Metabolome combined with transcriptome profiling reveals the dynamic changes in flavonoids in red and green leaves of Populus × euramericana ‘Zhonghuahongye’
Source: Front Plant Sci. 2023 Dec 21;14:1274700. doi: 10.3389/fpls.2023.1274700 (PMC10764563; doi:10.3389/fpls.2023.1274700)

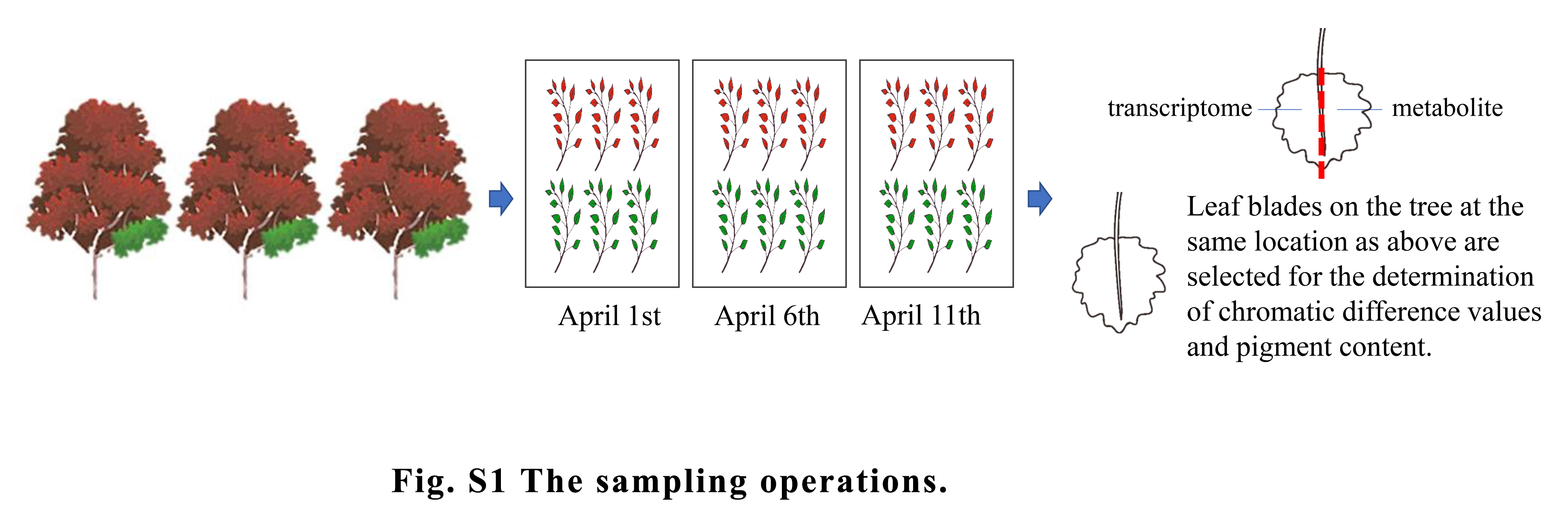

Supplement: Supplementary Figure 1 — The sampling operations. [file Image_1.tif]

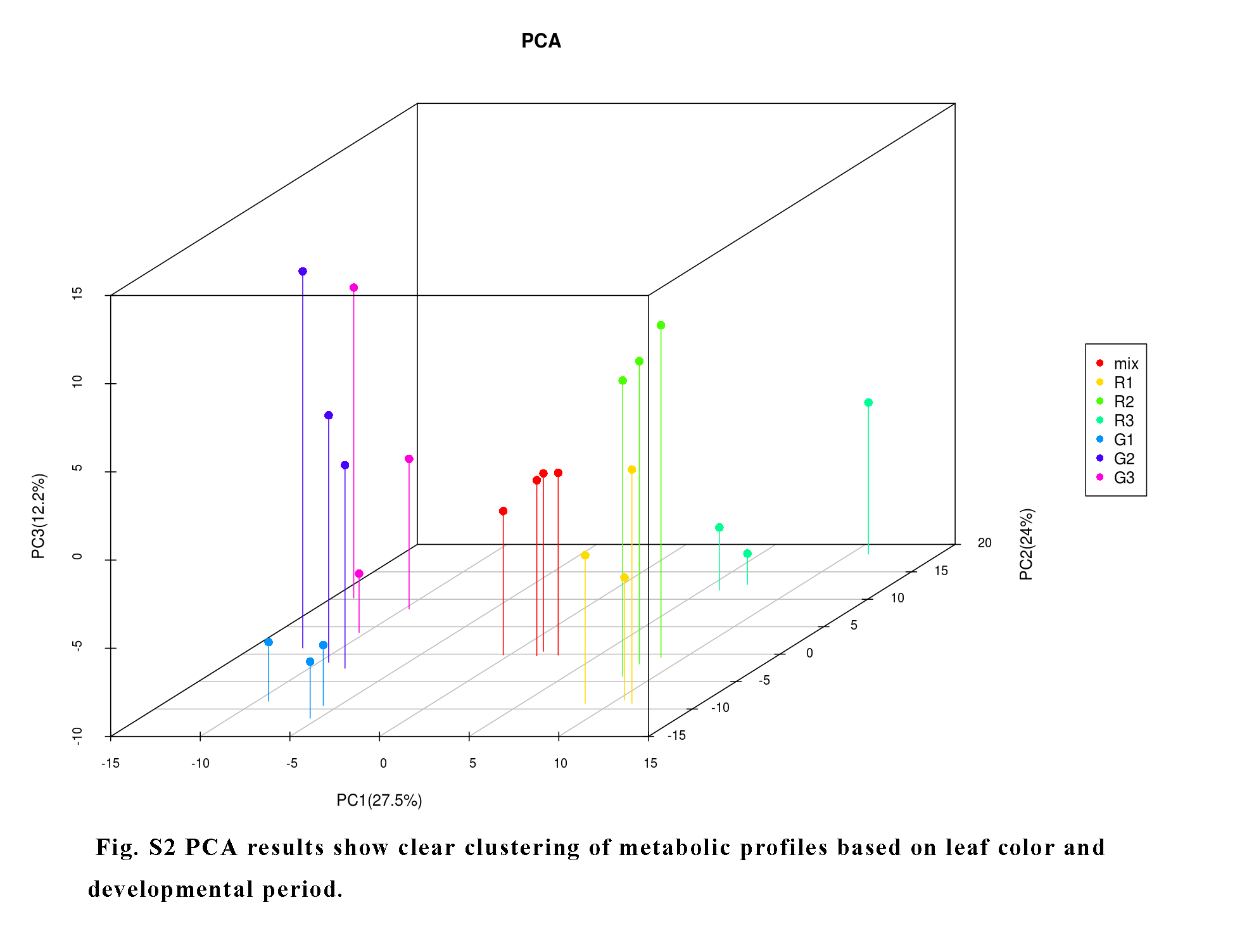

Supplement: Supplementary Figure 2 — PCA results show a clear clustering of metabolic profiles based on leaf color and developmental period. [file Image_2.tif]

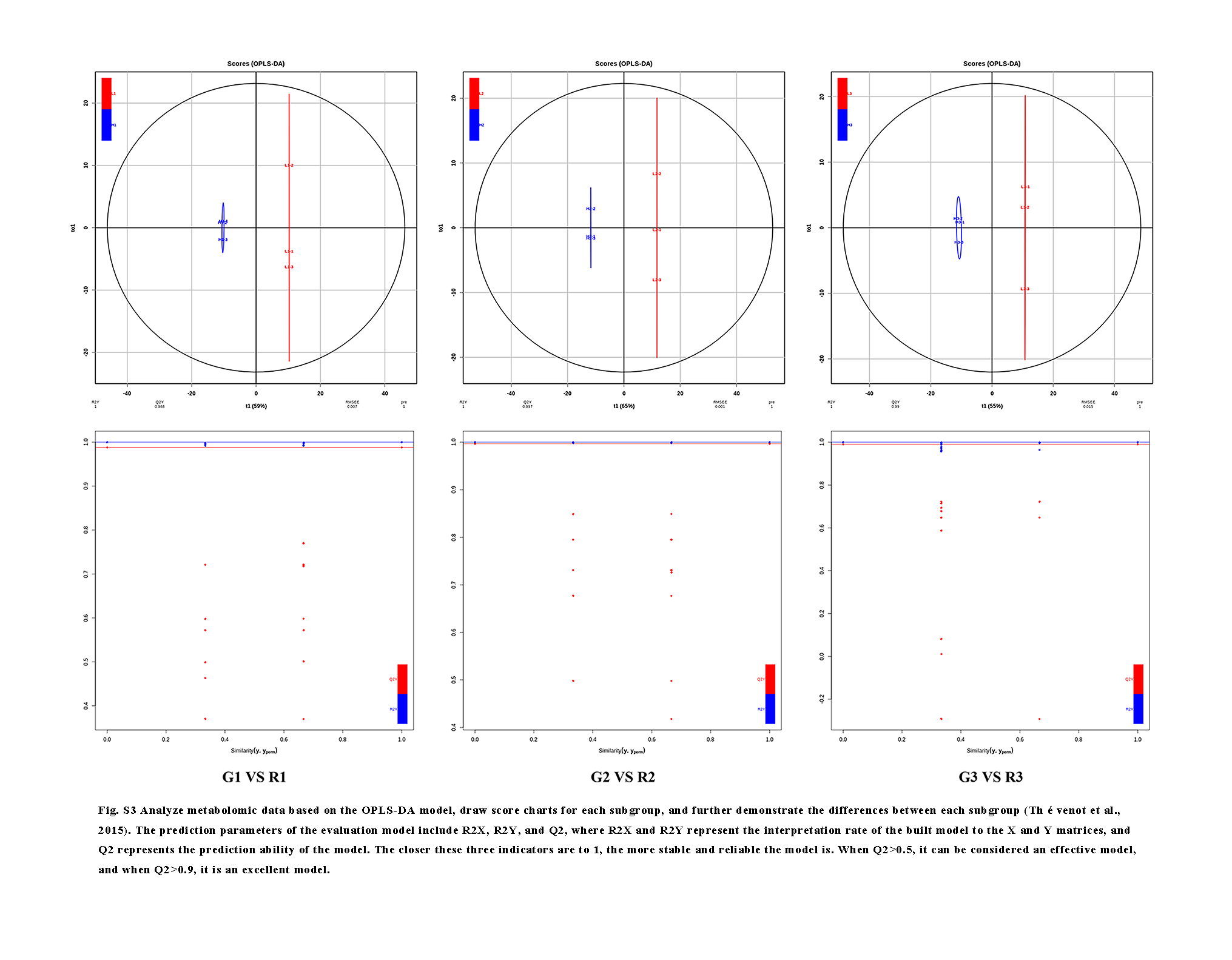

Supplement: Supplementary Figure 3 — Metabolomic data were analyzed based on the OPLS-DA model. [file Image_3.tif]

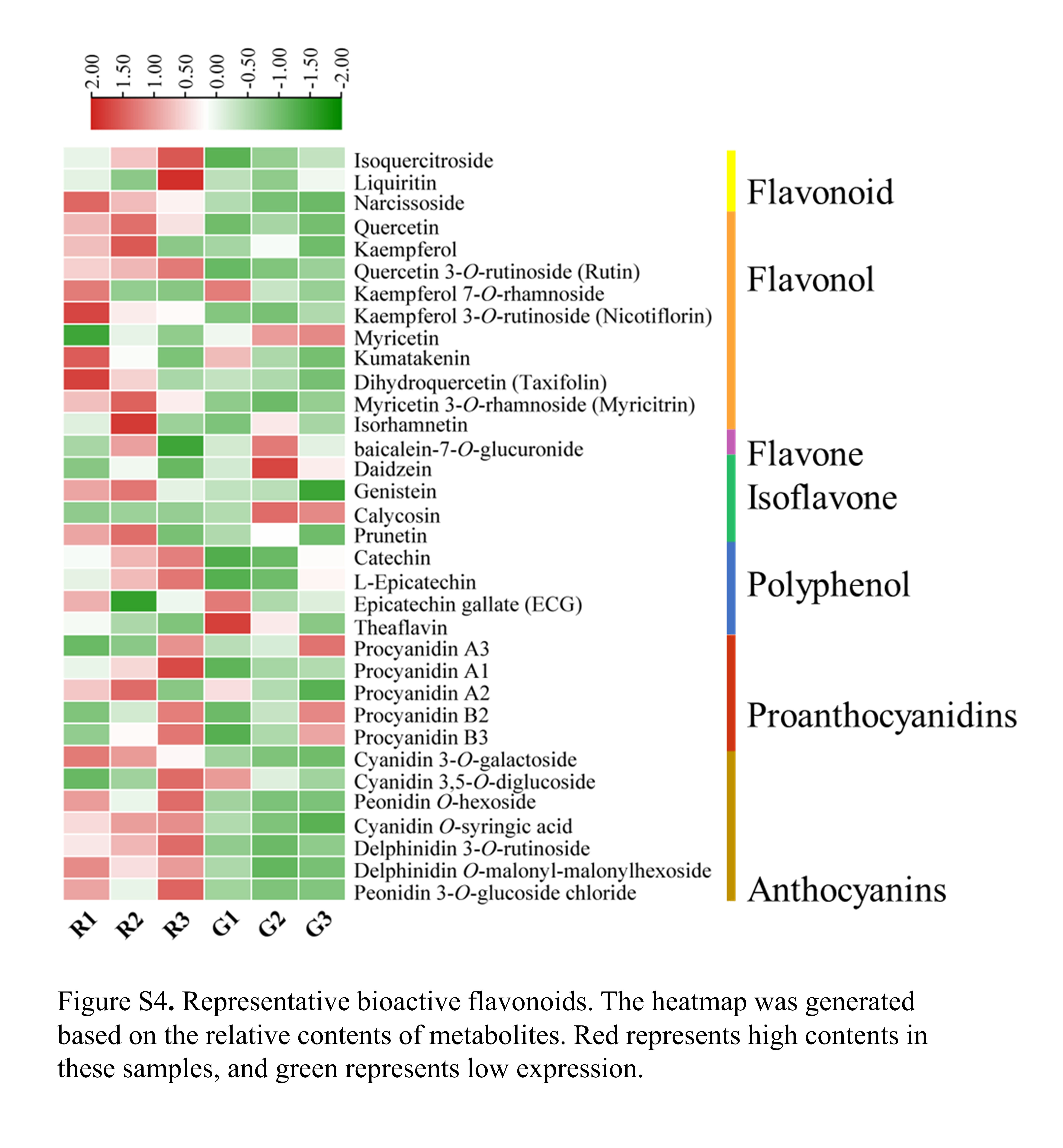

Supplement: Supplementary Figure 4 — Representative bioactive flavonoids. The heatmap was generated based on the relative contents of metabolites. Red represents high abundance in these samples, and green represents low abundance. [file Image_4.tif]

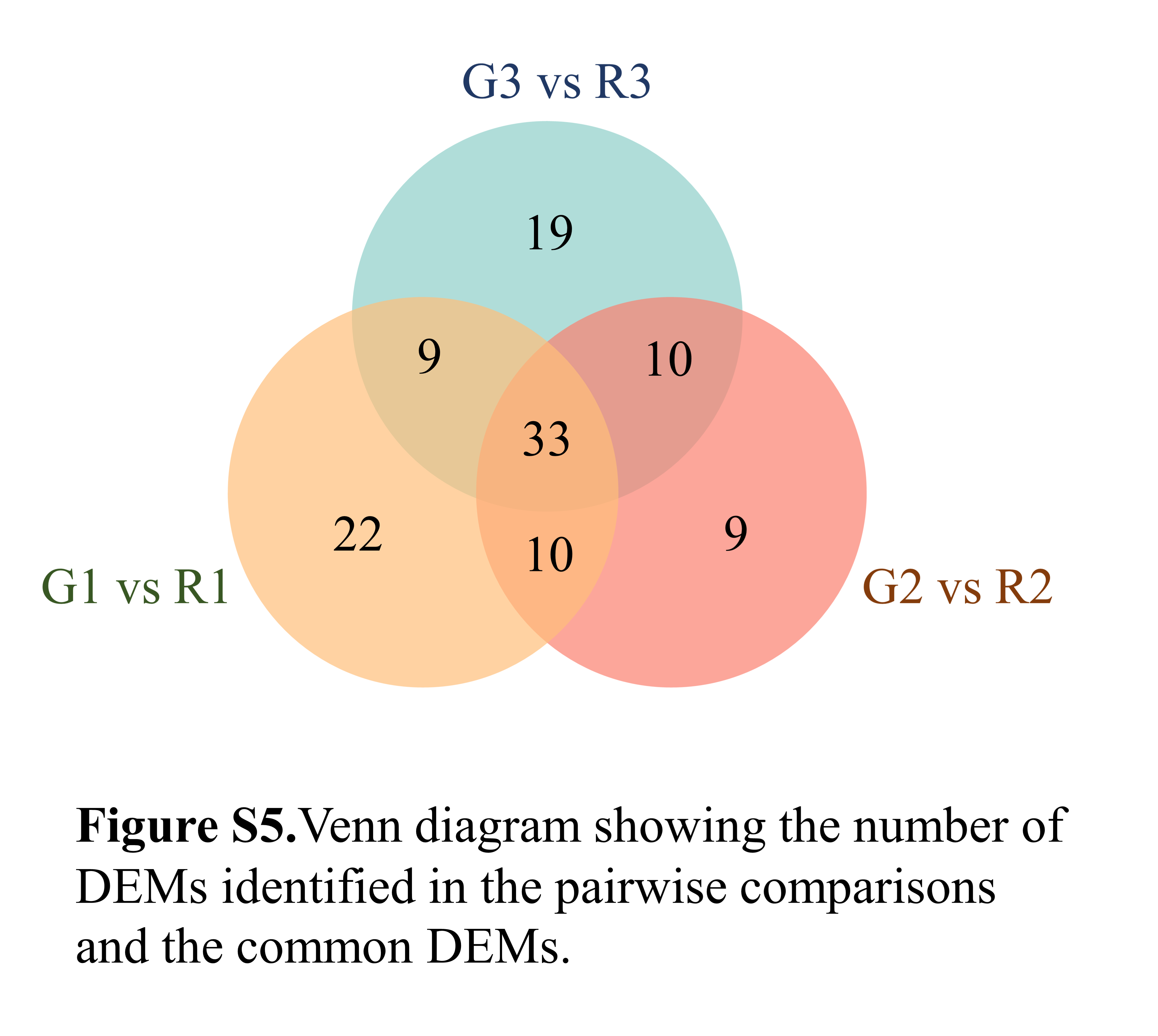

Supplement: Supplementary Figure 5 — Venn diagram showing the number of DEMs identified in the pairwise comparisons and the common DEMs. [file Image_5.tif]

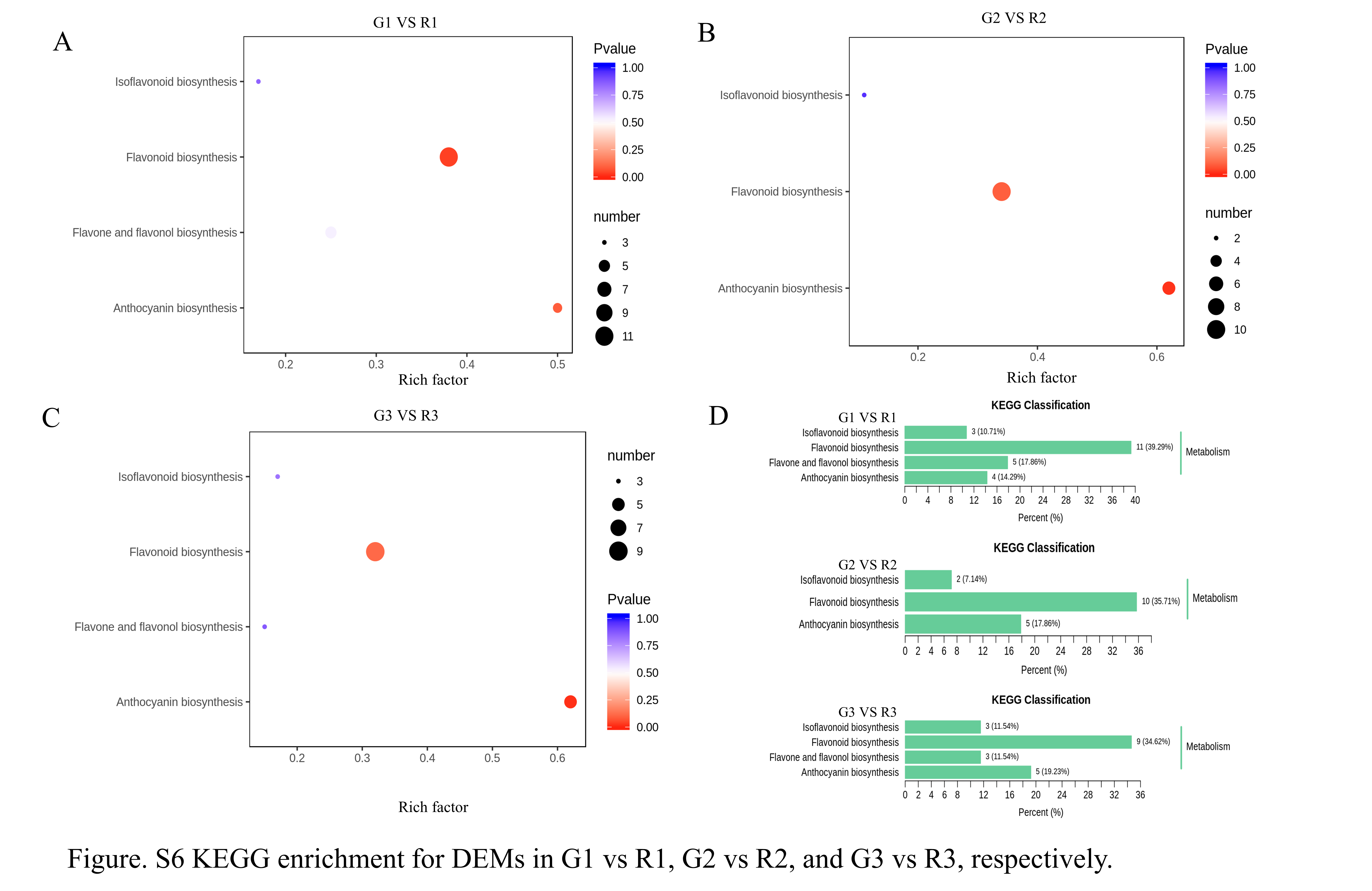

Supplement: Supplementary Figure 6 — KEGG enrichment bubble plots for DEMs in G1 vs. R1, G2 vs. R2, and G3 vs. R3. [file Image_6.tif]

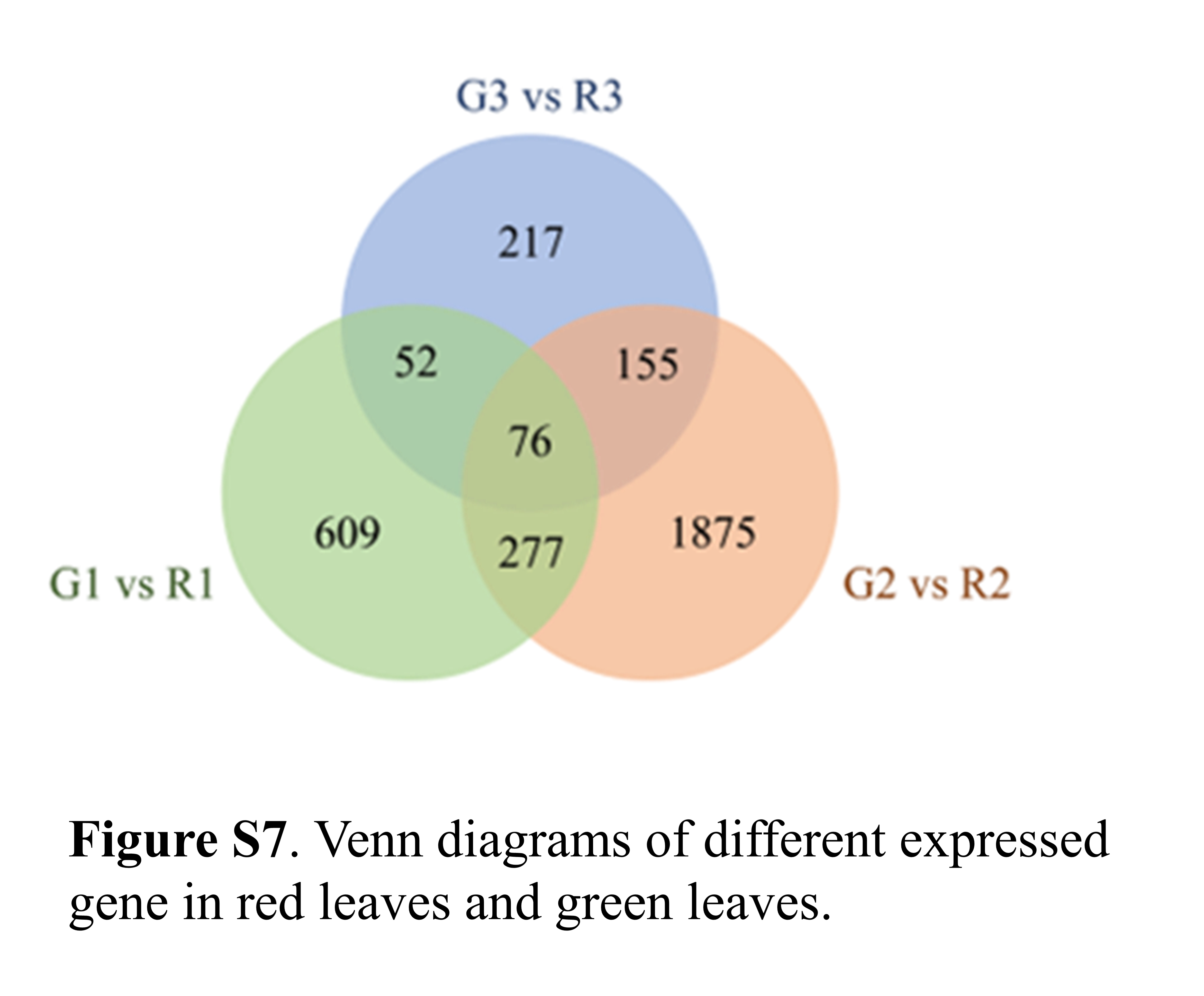

Supplement: Supplementary Figure 7 — Venn diagrams of differentially expressed genes in red leaves and green leaves. [file Image_7.tif]

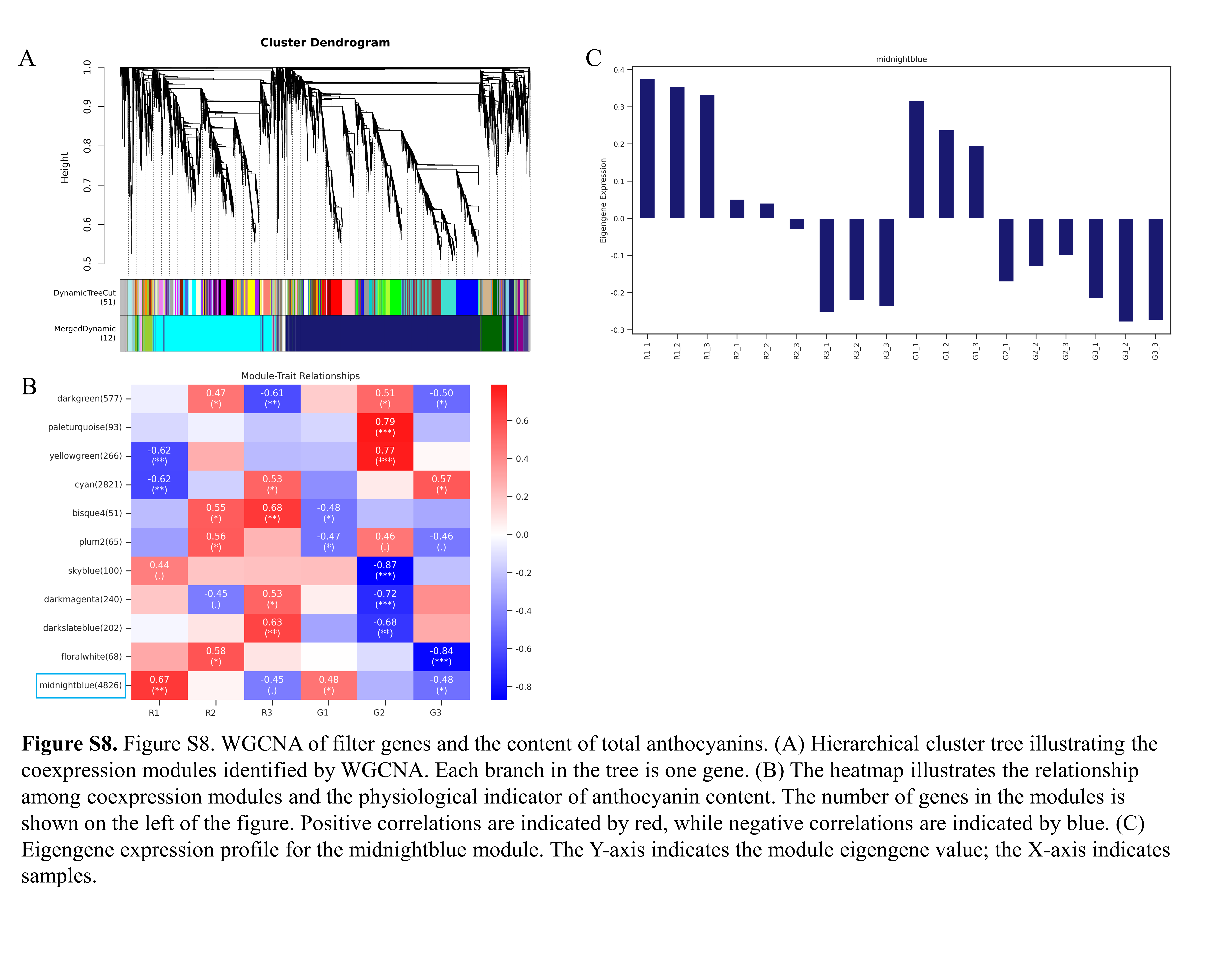

Supplement: Supplementary Figure 8 — WGCNA of filter genes and the total anthocyanin content. [file Image_8.tif]

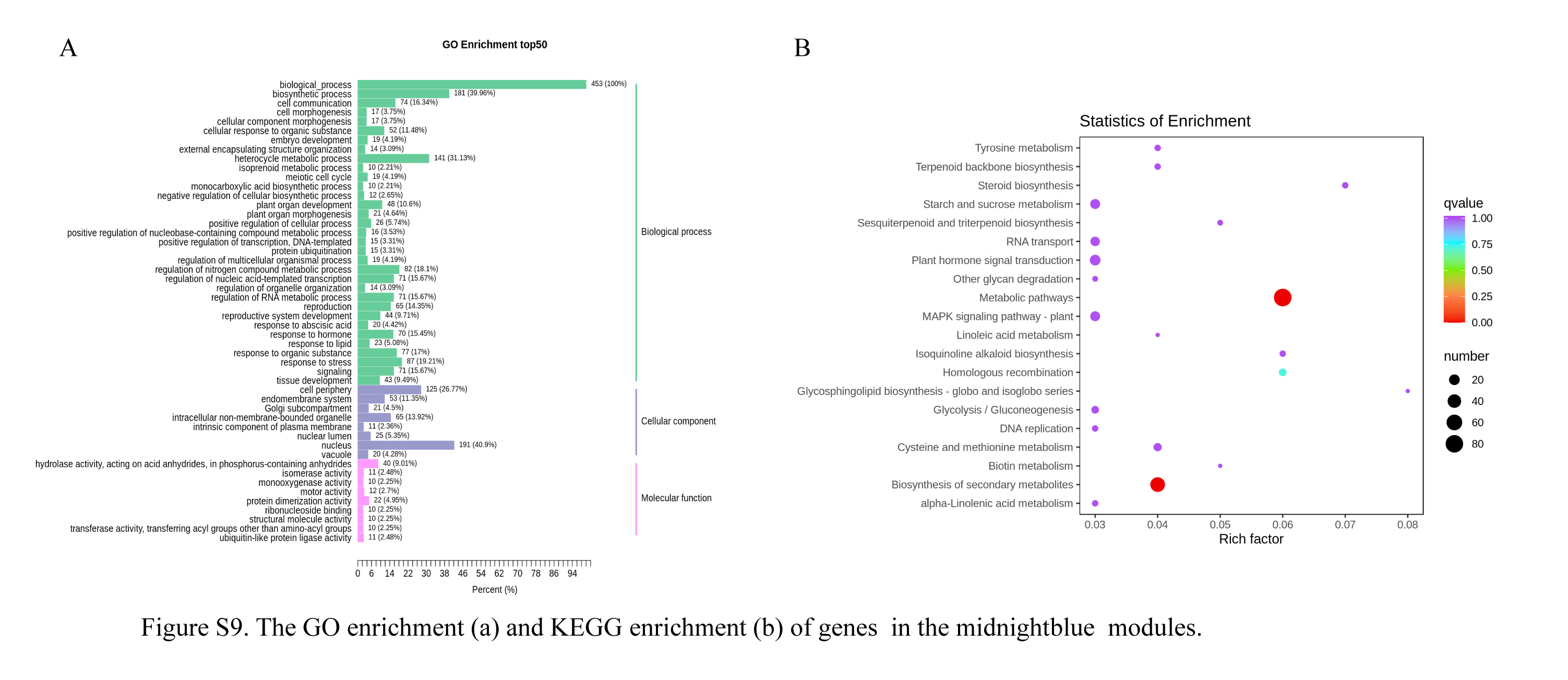

Supplement: Supplementary Figure 9 — The GO and KEGG enrichment of genes in the midnightblue modules. [file Image_9.tif]

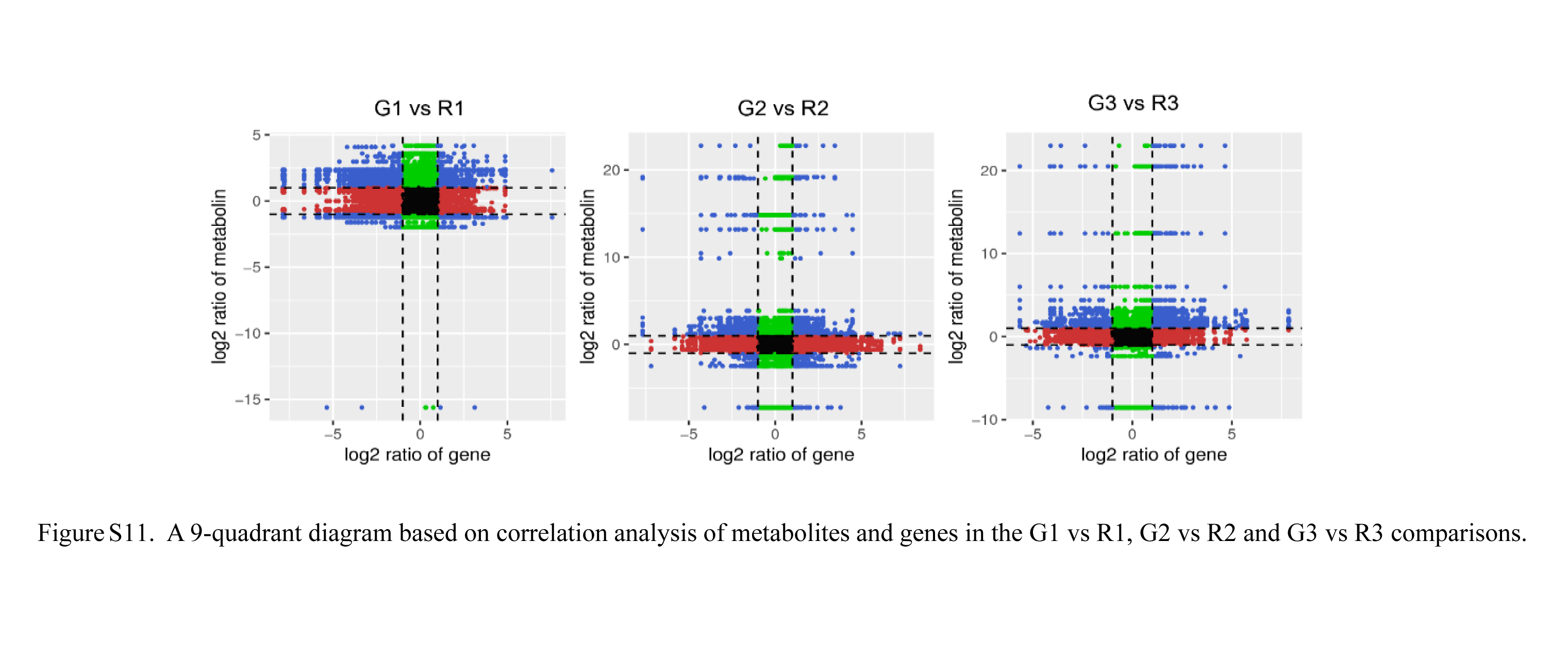

Supplement: Supplementary Figure 10 — A 9-quadrant diagram based on correlation analysis of metabolites and genes in the G1 vs. R1, G2 vs. R2, and G3 vs. R3 comparisons. [file Image_10.tif]

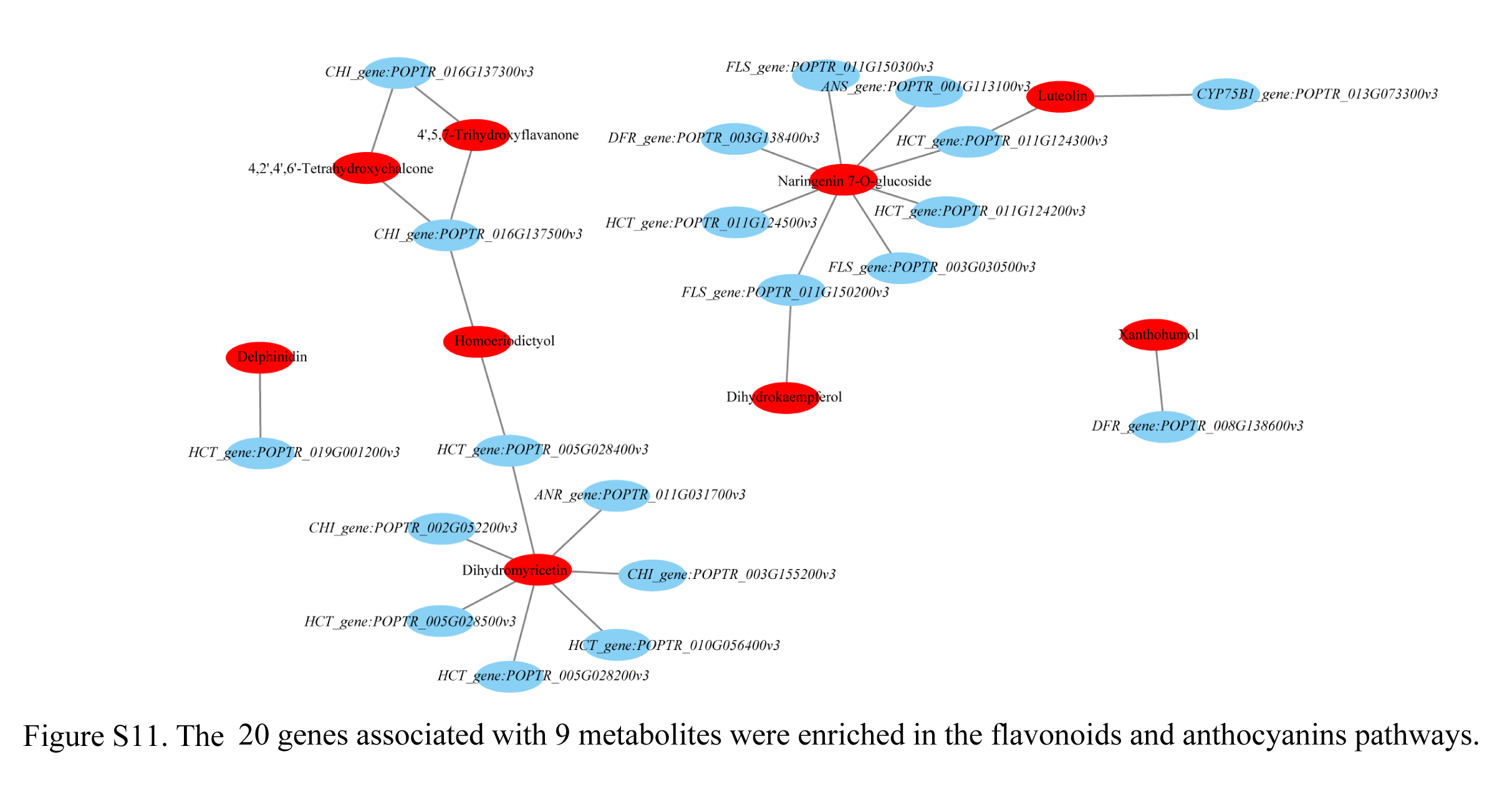

Supplement: Supplementary Figure 11 — The 20 genes associated with 9 metabolites were enriched in the flavonoid and anthocyanin pathways. [file Image_11.tif]

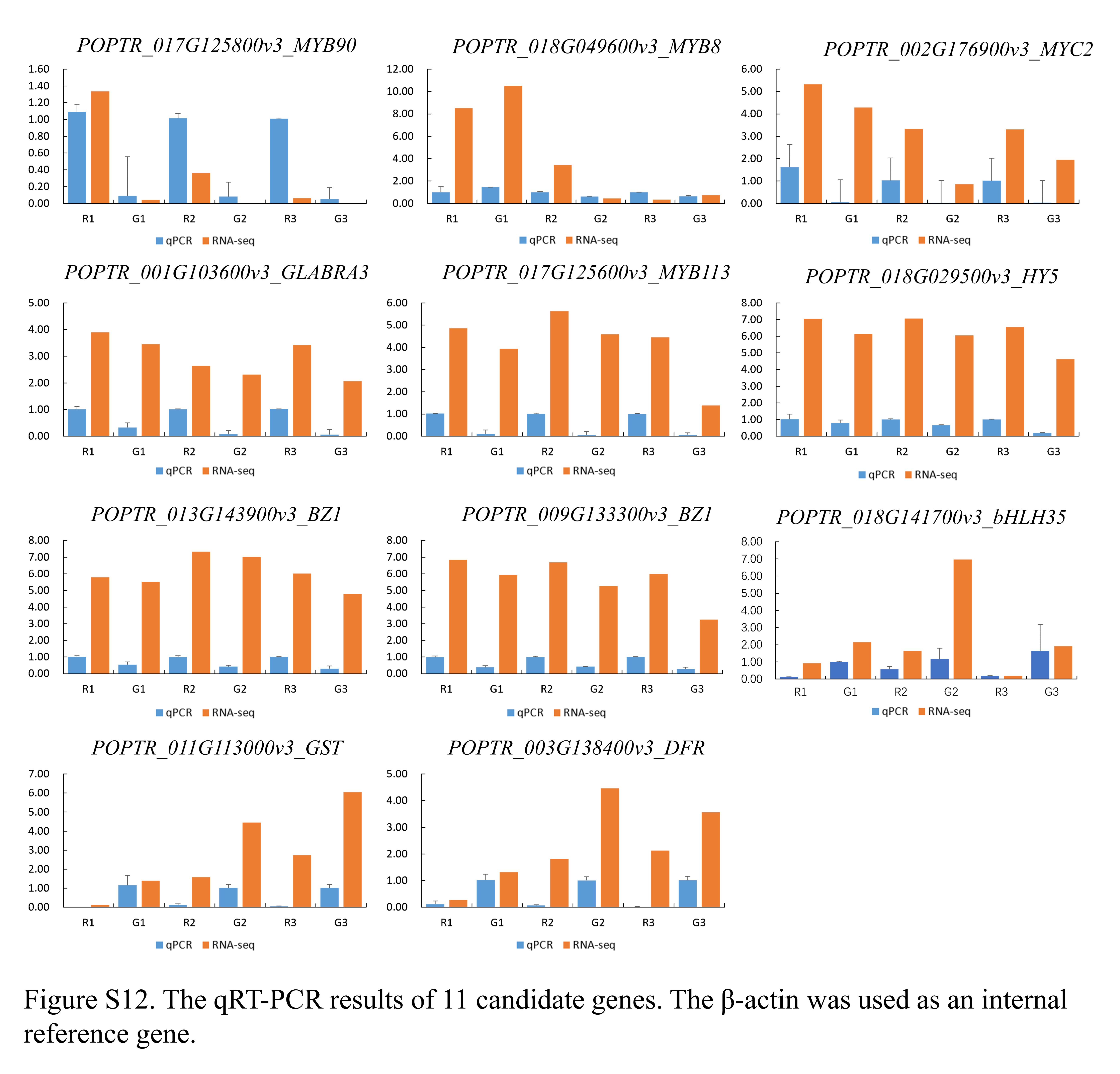

Supplement: Supplementary Figure 12 — qRT−PCR results of 11 candidate genes. β-Actin was used as an internal reference gene. [file Image_12.tif]
